# Cannabinoid exposure as a major driver of pediatric acute lymphoid Leukaemia rates across the USA: combined geospatial, multiple imputation and causal inference study

**DOI:** 10.1186/s12885-021-08598-7

**Published:** 2021-09-03

**Authors:** Albert Stuart Reece, Gary Kenneth Hulse

**Affiliations:** 1 Division of Psychiatry, University of Western Australia, Crawley, Western Australia 6009 Australia; 2 School of Medical and Health Sciences, Edith Cowan University, Joondalup, Western Australia 6027 Australia

**Keywords:** Acute lymphoid leukaemia, Childhood, Cannabis, Socioeconomic, Chromosomes, Genotoxicity, Epigenotoxicity

## Abstract

**Background:**

Acute lymphoid leukaemia (ALL) is the commonest childhood cancer whose incidence is rising in many nations. In the USA, between 1975 and 2016, ALL rates (ALLRs) rose 93.51% from 1.91 to 3.70/100,000 <  20 years. ALL is more common in Caucasian-Americans than amongst minorities. The cause of both the rise and the ethnic differential is unclear, however, prenatal cannabis exposure was previously linked with elevated childhood leukaemia rates. We investigated epidemiologically if cannabis use impacted nationally on ALLRs, its ethnic effects, and if the relationship was causal.

**Methods:**

State data on overall, and ethnic ALLR from the Surveillance Epidemiology and End Results databank of the Centre for Disease Control (CDC) and National Cancer Institute (NCI) were combined with drug (cigarettes, alcoholism, cannabis, analgesics, cocaine) use data from the National Survey of Drug Use and Health; 74.1% response rate. Income and ethnicity data was from the US Census bureau. Cannabinoid concentration was from the Drug Enforcement Agency Data. Data was analyzed in R by robust and spatiotemporal regression.

**Results:**

In bivariate analyses a dose-response relationship was demonstrated between ALLR and Alcohol Use Disorder (AUD), cocaine and cannabis exposure, with the effect of cannabis being strongest (β-estimate = 3.33(95%C.I. 1.97, 4.68), *P* = 1.92 × 10^− 6^). A strong effect of cannabis use quintile on ALLR was noted (Chi.Sq. = 613.79, *P* = 3.04 × 10^− 70^). In inverse probability weighted robust regression adjusted for other substances, income and ethnicity, cannabis was independently significant (β-estimate = 4.75(0.48, 9.02), *P* = 0.0389). In a spatiotemporal model adjusted for all drugs, income, and ethnicity, cannabigerol exposure was significant (β-estimate = 0.26(0.01, 0.52), *P* = 0.0444), an effect increased by spatial lagging (THC: β-estimate = 0.47(0.12, 0.82), *P* = 0.0083). After missing data imputation ethnic cannabis exposure was significant (β-estimate = 0.64(0.55, 0.72), *P* = 3.1 × 10^− 40^). 33/35 minimum e-Values ranged from 1.25 to 3.94 × 10^36^ indicative of a causal relationship. Relaxation of cannabis legal paradigms had higher ALLR (Chi.Squ.Trend = 775.12, *P* = 2.14 × 10^− 112^). Cannabis legal states had higher ALLR (2.395 ± 0.039 v. 2.127 ± 0.008 / 100,000, *P* = 5.05 × 10^− 10^).

**Conclusions:**

Data show that ALLR is associated with cannabis consumption across space-time, is associated with the cannabinoids, THC, cannabigerol, cannabinol, cannabichromene, and cannabidiol, contributes to ethnic differentials, demonstrates prominent quintile effects, satisfies criteria for causality and is exacerbated by cannabis legalization.

**Supplementary Information:**

The online version contains supplementary material available at 10.1186/s12885-021-08598-7.

## Background

Acute lymphoid leukaemia (ALL) is the commonest childhood cancer and accounts for about 25% of all childhood cancer cases [1]. In 2012 globally there were 352,000 cases of leukaemia with 265,000 deaths [2]. ALL has undergone a significant and unexplained increase in many nations, with global incidence and mortality rates of leukaemia of 4.7 and 3.4 / 100,000 closely aligned due its high mortality in developing nations [2]. Across Europe from 1978 to 1997 the ALL rate (ALLR) rose from 3.2 to 3.7 / 100,000 (16.9%) in children, and from 0.85 to 1.2 (44.7%) in adolescents, being markedly worse in the north [3]. In New Zealand from the period 1968–1972 to the period 1988–1990 the age standardized incidence of ALL rose from 4.83 to 7.04 / 100,000 (45.7%) [4].

Marked ethnic disparities have also been reported in New Zealand with the rate in patients of Caucasian background being 3.2 / 100,000 compared to only 1.3 / 100,000, or 40.6% less in those of Maori background. Similarly, in the USA long term data series from 1975 to 2017 show that the overall ALLR has risen markedly and is more prevalent in Americans of Caucasian background. Data on the Surveillance Epidemiology and End Results (SEER) Explorer website reveal that at the national level the age-adjusted ALLR for all ethnicities and all stages in ages < 20 years rose from 1.9124 / 100,000 in 1975 to 3.7007 / 100,000 in 2016, a 93.51% rise. The age-adjusted modelled ALLRs in patients < 20 years rose from 2.50 to 3.45 (/100,000 or 37.4%) 1975–2017 [5]. For the period 2000–2017 this is listed as a 0.6% annual percent change which is highly significant (*P* < 0.01). Nationally the mean rate in patients of Caucasian-American background is 3.75 ± 0.76 compared to 2.23 ± 0.77 (mean ± S.E.M.) in patients of African-American background (t = 14.42, df = 53.14, *P* = 2.60 × 10^− 20^) [5].

The cause of this rise both in the USA and globally is unknown, as is the aetiology of the marked ethnic differences.

### Acute lymphoid leukaemia

One clue may be the widespread recognition that most pediatric cancers arise during in utero life as a result of genetic or epigenetic errors [6, 7]. Amongst other factors reports exist of a link between prenatal cannabis exposure (PCE) and other leukaemia’s, including acute myeloid leukaemia and acute myelomonocytic leukaemia [8, 9] although this association has been contested [10]. Accordingly, investigators have looked for a similar association with ALL with negative results [8–10], however outcomes may been confounded in earlier studies by ALL incidents requiring a threshold level of cannabis exposure. As cannabis use has risen globally since 1975, including use by pregnant women or females of reproductive age, the possibility of cannabis as a driver of these dual mysterious trends bears serious consideration.

National Survey of Drug Use and Health (NSDUH) data from the Substance Abuse and Mental Health Services Administration (SAMHSA) reveal that between 2016 and 2019 the number of Americans estimated to have used cannabis in the prior month rose in the three age categories 17–21 years, 18–25 years and > 26 years from 6.5 to 7.4%, from 20.8 to 23.0% and from 7.2 to 10.2% respectively, representing total rises from 24 million to 31.5 million or 31.25% elevation across those three years [11]. In 2017 161,000 American women were estimated to have smoked cannabis while pregnant [11]. In a 2018 published study 69% of Colorado dispensaries contacted advised pregnant women that cannabis use during pregnancy was safe [12], despite the American College of Obstetricians and Gynaecologists (ACOG) and the American Academy of Pediatrics (AAP) recommending otherwise [13–17]. 24% of pregnant Californian teenagers recently tested positive to cannabis [18].

Whilst much of the debate relating to cannabis centres on its main psychoactive component tetrahydrocannabinol (THC), it is important to note that other cannabinoids have also been implicated in carcinogenic pathways. For example THC, cannabinol and cannabidiol have been implicated in end to end chromosomal translocations [19] and cannabidiol and is propyl ester cannabidivarin, in low micromolar doses, have been shown to induce DNA double strand breaks, induce micronucleus formation and directly oxidize both the purine and pyrimidine bases of DNA [20].

The present study examined if the previously described link between prenatal cannabis exposure and childhood leukaemia: (1) could be extended to ALL with different analytical strategies; (2) was observable and salient at the population health level; (3) linked in a space-time analysis with trends of cannabis use; (4) might be a driver of the recent rise in ALL; (5) might account for some of the variance related to the known and described ALLR by ethnic background; and (6) whether the relationship satisfied the quantitative formal criteria of causality. USA data was selected as both drug use and ALLRs by state and year, along with other important covariates, were publicly available.

## Methods

### Data

Age-adjusted ALLR for all patients < 20 years and by selected ethnicities were downloaded via the SEERStat Software from the National Program of Cancer Registries (NPCR) and Surveillance Epidemiology and End Results (SEER) Incidence File from the US Cancer Statistics Public Use Database, Submission 2001–2017 [21]. National rates were taken from the SEER*Explorer website of the Centers for Disease Control Atlanta Georgia (CDC) and National Cancer Institute [5]. Drug use by US state was taken from the Restricted Use Data Analysis System (RDAS) of the Substance Abuse and Mental Health Data Archive (SAMHDA) of the National Survey of Drug Use and Health (NSDUH) from the Substance Abuse and Mental Health Services Administration (SAMHSA) for the period 2003–2017 [22]. The drugs of interest were monthly cigarette use (Cigarettes), Alcohol Use Disorder (AUD), last month cannabis use (Cannabis), last year analgesic misuse (Analgesics), and last year cocaine use (Cocaine). State median household income and ethnicity data was downloaded via the tidycensus package within R [23] from the US Census bureau. The ethnicities of interest were Caucasian-American, African-American, Asian-American, Hispanic-American, American Indian / Alaskan Native (AIAN)-American and Native Hawaiian-Pacific Islander (NHPI) – American. The THC concentration in Federal seizures was derived from publications from the Drug Enforcement Agency [24–26]. Data on the legal status of cannabis by state was taken from an internet search [27].

### Derived data

The SAMHDA RDAS lists a variable at the national level called mrjmdays, which provides data on the intensity of cannabis use by ethnicity in the month prior to the survey in the categories 0 days, 1–2 days, 3–5 days, 6–19 days, 20–30 days. In each year of the NSDUH this variable can be summed by ethnicity so that the percentage using cannabis in each category can be summed to provide an index of the intensity of cannabis use at the Federal level for that ethnicity. These ethnic scores were then multiplied by the state rates of last month cannabis use and by the concentration of THC in Federal seizures to derive an estimate of ethnic THC exposure at the state level. State rates of cannabinoid exposure were derived by multiplying the concentration of the cannabinoid identified in Federal seizures by the mean rate of last month cannabis use in that state. Quintiles of cannabis use were calculated for each year by dividing states into five groups based on their surveyed last month cannabis use rates.

### Statistics

Data was processed using R version 4.0.2 and R-Studio 1.3.1093 in October 2020 [28]. Data was pre-processed using the dplyr and tidycensus packages [23, 29]. Point estimates are listed as mean + standard error of the mean. Data were log-transformed guided by the Shapiro-Wilks test. Graphs were drawn in in R-Base, ggplot2 and lattice [28–30]. Maps were drawn in sf and ggplot2 [29, 31, 32]. Correlograms were drawn in corrplot and corrgram [33, 34]. Initial regression models were manually reduced in the classical manner by serial deletion of the least significant term to adduce final models. Two by two epidemiological table analysis was with epiR version 2.0.11 [35].

Several regression model forms were used in order to harness the various strengths of each model type. Straightforward linear regression was performed by linear regression. Mixed effects regression was used to capture the serial repeated nature of the data, to utilize inverse probability weights and to provide standard deviations for e-value calculations. Panel regression was performed as the data were inherently of that type, models could accept missing values, instrumental variables could be utilized, models could be temporally lagged and models could utilize inverse probability weights. However panel models do not accept data with repeated space-time indices as required in ethnicity studies and do not provide model standard deviations. Spatial regression was performed as data were inherently spatiotemporally distributed; spatial model coefficients confirmed the importance and significance of considering the spatiotemporal distribution of data; both spatial and temporal lagging could be conducted; and standard deviations could be calculated from which to calculate e-values; however inverse probability weights are not accepted, instrumental variables cannot be used and missing values are not tolerated. Robust regression was performed both to utilize inverse probability weights and to provide robust regression estimates, but standard deviations cannot be calculated from such models nor instrumental variables or lagging used.

Linear regression was conducted in R-base. Fitted values were calculated from the model objects. Extension of the time series of linear models was performed using the predict function from package stats [28]. Robust regression was conducted with the survey package [36]. Repeated measures mixed effects regression was conducted with package nlme [37] using State as a random effect. Inverse probability weights were constructed using package ipw [38]. Panel regression was calculated using package plm [39]. E-Values were calculated using package EValue [40].

Spatial neighbour relationships were constructed in spdep [41] and spatiotemporal regression was conducted in splm [42] with the spatial panel random effects maximum likelihood (spreml) function [43]. Model specification and error structure was determined using the final model regression coefficients from a full model including serial correlation in the remainders, spatial error effects after Kelejian, Kapoor and Prucha [44], spatial lag effects and random effects (sem2srre) and only utilizing those effects which were significant as recommended [45].

Multiple imputation by chained equations for ethnicity data was conducted in R-package mice [46]. As 47.94% of the state-level ethnicity data was missing 60 imputations with 60 iterations each were conducted following Van Buuren and Groothius-Oudshoorn [46, 47]. Imputation was performed by the classification tree (“cart”) method which provided the best ethnic-specific ALLRs and resulted in fractions of missing data of only 3.1% in simple linear models regressing ALLR against cannabis exposure. All interactions were calculated prior to data imputation. Linear models were calculated on each imputed dataset and the pooled results were combined in accordance with Rubin’s rules [46, 47].

All t-tests were two sided. *P* < 0.05 was considered significant.

### Data sharing and availability

Data including software code in R has been made freely available through the Mendeley data repository at this URL 10.17632/cf8c43yv62.1 .

### Ethics

This study received ethical approval from the University of Western Australia Human Research Ethics Committee on 7th January 2020 RA/4/20/7724.

## Results

Data from the SEER*Explorer website reveal that the annual age-adjusted modelled incidence of pediatric ALL climbed significantly from 2.4970 to 3.4513 / 1,000,001,975–2017 across all races and all stages combined which represents an 0.7736 annual percent rise. Amongst Caucasian-Americans the modelled age-adjusted rate rose from 2.6495 to 3.8150 / 100,000 across this same period. No modelled rates are listed on the SEER*Explorer site for ethnic minorities. 50.89% of cases occurred in those younger than 20 years.

The NSDUH advises that it has a 74.1% response rate [48].

Age-adjusted ALLRs by state were downloaded from the SEER database 2001–2017 as described. States with less than 15 cases are routinely suppressed. Complete datasets are required for spatiotemporal analysis as techniques do not accommodate missing data. Data from 31 states was complete. Data from Idaho, Mississippi and Nebraska was incomplete and was completed by temporal kriging. The missing data rate was 14 cases from 576 cases or 2.4%. The complete kriged dataset is shown in Supplementary Table 1 with imputed data marked.

**Fig. 1:**
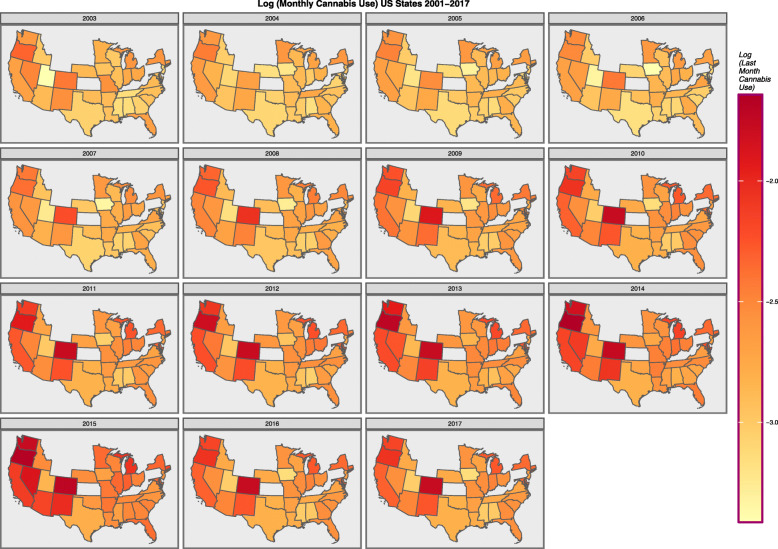
Choropleth Map of age-adjusted ALL rates across USA

Figure 1 illustrates this data across the USA map-graphically for log ALLR’s.

**Fig. 2:**
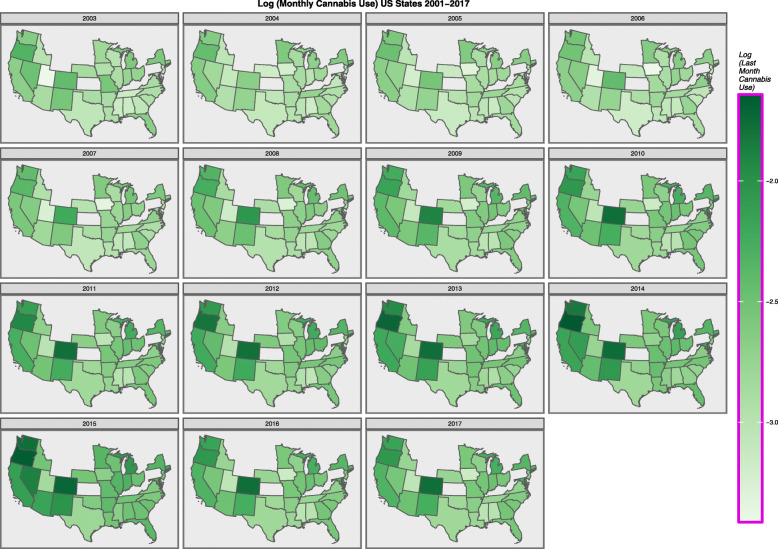
Choropleth Map of age-adjusted last month cannabis use rates across USA (NSDUH, SAMHSA data)

Figure 2 shows the log rate of last month cannabis use map-graphically across the USA.

**Fig. 3:**
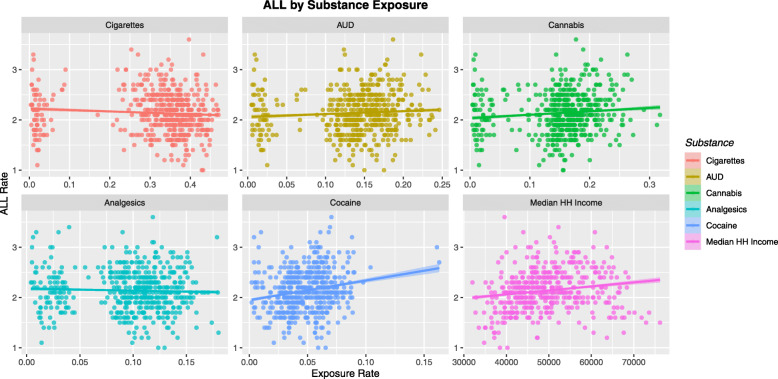
ALL rate by Substance Use

Figure 3 shows the ALLR as a function of the various substances used in the community. Rising trends are noted with AUD, cannabis, cocaine and median household income.

**Fig. 4:**
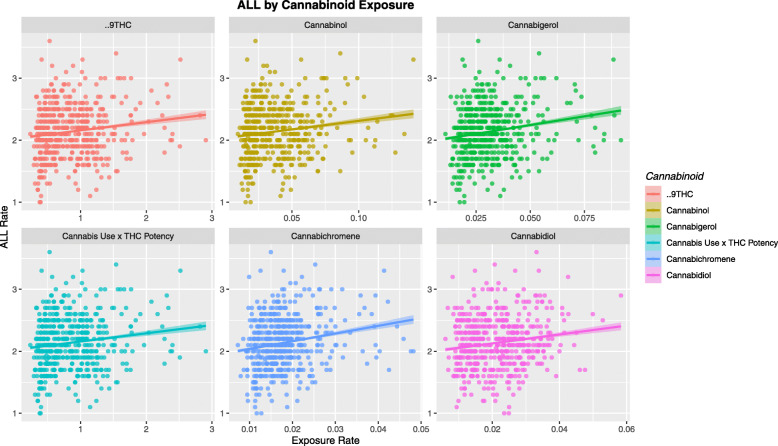
ALL rate by Cannabis / Cannabinoid use

Figure 4 shows the ALLR as a function of exposure to the cannabinoids THC, cannabinol, cannabigerol, cannabichromene and cannabidiol.

**Fig. 5:**
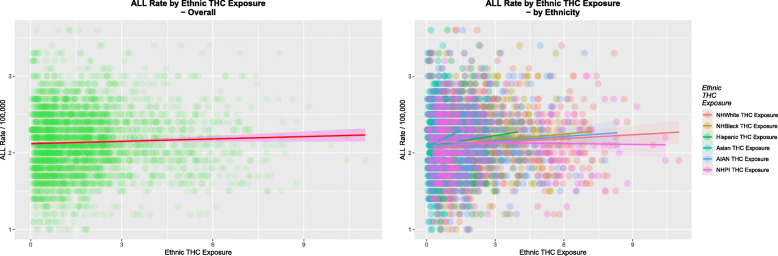
ALL rate by Ethnic THC Exposure (**A**) Overall and (**B**) by Ethnicity

Figure 5 shows the ALLR as a function of the ethnic THC exposure for all ethnicities (A) together and for (B) each of the ethnicities of interest.

Table 1 lists the various regression slopes of these lines together with their significance levels. The slope for the cannabis use line is noted to be highly significant (β-estimate = 3.33, (95%C.I. 1.97–4.68), *P* = 1.92 × 10^− 6^). The slopes of all of the regression lines for the cannabinoids cannabichromene, cannabigerol, cannabinol and cannabidiol were also significant. Log transformation improved the normality compliance of these data so these results are also listed.

**Table 1:** Introductory linear regression results

Parameter	Parameter	
	Estimate (C.I.)	***P***-Value
***Substances***		
Cannabis	3.33 (1.97, 4.68)	1.9E-06
AUD	4.32 (1.9, 6.75)	5.2E-04
Cocaine	7.93 (2.3, 13.57)	0.0060
Analgesics	1.88 (− 1.93, 5.7)	0.3340
Cigarettes	−2.78 (−3.59, −1.96)	5.6E-11
***Income***		
Income	0.41 (0.21, 0.61)	6.7E-05
***Cannabinoids***		
Cannabis	3.33 (1.97, 4.68)	1.9E-06
CBC Exposure	12.42 (6.94, 17.91)	1.1E-05
CBG Exposure	5.65 (2.92, 8.37)	5.6E-05
CBD Exposure	7.19 (3.1, 11.29)	6.2E-04
CBN Exposure	2.76 (1.13, 4.38)	9.5E-04
THC Exposure	0.13 (0.05, 0.21)	0.0012
***Log (Cannabinoids)***		
log (Cannabis)	0.3 (0.18, 0.41)	4.3E-07
log (CBC Exposure)	0.27 (0.16, 0.38)	2.2E-06
log (CBG Exposure)	0.22 (0.12, 0.31)	1.0E-05
log (THC Exposure)	0.14 (0.06, 0.21)	2.6E-04
log (CBN Exposure)	0.12 (0.05, 0.19)	4.3E-04
log (CBD Exposure)	0.13 (0.05, 0.22)	0.0023
***Ethnicity***		
log (Hispanic-American)	0.27 (0.23, 0.31)	< 2.2E-16
log (Asian-American)	0.17 (0.12, 0.22)	2.0E-10
log (NHPI-American)	0.11 (0.08, 0.15)	9.5E-10
asinh (AIAN-American)	3.45 (1.86, 5.04)	2.6E-05
log (Caucasian-American)	−0.33 (− 0.51, − 0.14)	5.1E-04
log (Black-American)	− 0.16 (− 0.2, − 0.13)	< 2.2E-16
***Ethnic THC Exposure***		
log (Asian THC Exposure)	0.08 (0.03, 0.13)	0.0025
log (Hispanic THC Exposure)	0.08 (0.03, 0.13)	0.0033
log (NHBlack THC Exposure)	0.08 (0.03, 0.13)	0.0033
log (NHWhite THC Exposure)	0.08 (0.03, 0.14)	0.0041
log (AIAN THC Exposure)	0.07 (0.01, 0.13)	0.0172
log (NHPI THC Exposure)	0.02 (−0.02, 0.06)	0.2810

Importantly the slopes of all of the lines for ethnic THC exposure were positive and significant with the single exception of the NHPI-American ethnicity.

Table 2 lists the correlation matrix for these data, conflated in both cases with the relevant significance matrix. The upper top right in both cases shows the Pearson correlation coefficients and the lower bottom left half-matrix shows the applicable significance levels. Matrix A lists the various substances, income and ethnicities. Matrix B lists the results for ethnic THC exposure and cannabinoid exposure. Results are colour coded.

**Table 2:**
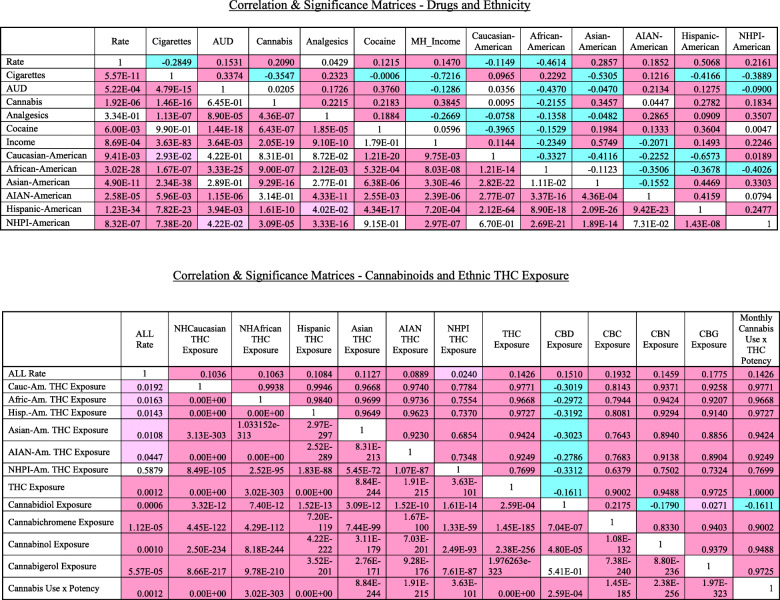
Correlation & significance matrices

**Fig. 6:**
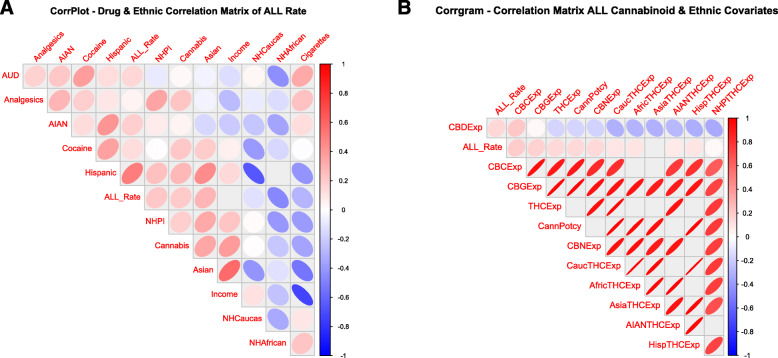
Corrplot correlogram (**A**) Drugs and ethnic correlations and (**B**) Cannabinoid and Ethnic THC Exposure correlations

**Fig. 7:**
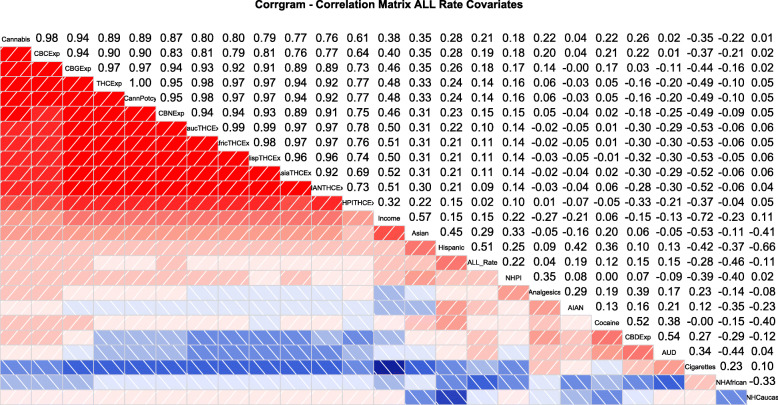
Corrgram correlogram for all variables

Similar results are listed graphically in the correlograms shown in Figs. 6 and 7 constructed with corrplot and corrgram respectively.

In Fig. 5 positive correlations are shown as red ellipses sloping upward and to the right. Stronger correlations are illustrated by narrower ellipses and the brighter tone shades. The positive association of the ALLR with substance and ethnic THC exposure is clear from these figures.

The corrgram correlogram shown in Fig. 7 has been ordered by hierarchical clustering for all covariates together. The ALLR is noted here to correlate with most cannabinoids, most ethnicities and most substances with the exception of cigarettes, Non-Hispanic Caucasian and Non-Hispanic African-American ethnicities.

Table 3 shows the quintile composition by state for each quintile of cannabis use.

**Table 3:** Cannabis use Quintiles by State

State	Quintile 1	Quintile 2	Quintile 3	Quintile 4	Quintile 5
Alabama	14	1	0	0	0
Arizona	0	3	7	5	0
Arkansas	0	6	9	0	0
California	0	0	0	15	0
Colorado	0	0	0	1	14
Connecticut	0	0	2	12	1
Florida	0	0	14	1	0
Georgia	2	3	8	2	0
Idaho	6	9	0	0	0
Illinois	0	2	13	0	0
Indiana	0	1	2	12	0
Iowa	14	1	0	0	0
Louisiana	7	8	0	0	0
Maryland	2	3	1	9	0
Michigan	0	0	0	15	0
Minnesota	0	3	7	4	1
Mississippi	14	1	0	0	0
Missouri	0	0	14	1	0
Nebraska	9	4	2	0	0
Nevada	0	0	4	9	2
New Jersey	8	7	0	0	0
New Mexico	0	0	0	15	0
New York	0	0	0	15	0
North Carolina	0	11	4	0	0
Ohio	0	0	13	2	0
Oklahoma	10	4	1	0	0
Oregon	0	0	0	0	15
South Carolina	4	6	5	0	0
Tennessee	1	8	4	2	0
Texas	15	0	0	0	0
Utah	15	0	0	0	0
Virginia	2	8	4	1	0
Washington	0	0	0	2	13
Wisconsin	0	14	1	0	0

**Fig. 8:**
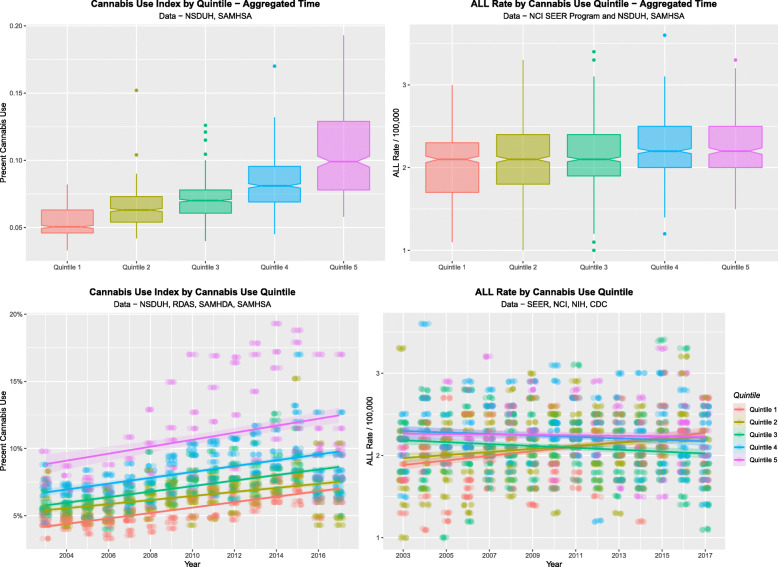
Quintile analysis of Cannabis use (**A** and **C)** and ALL Rates (**B** and **D**) by cannabis use quintile as time-dependent scatterplots (**B** and **D**) and boxplots (**A** and **C**)

Figure 8 shows a quintile analysis of the cannabis use data (A, C) alongside the ALLR (B, D) by cannabis use quintile as both scatterplots (B, D) over time and as boxplots over aggregated time (A, C). One reads the boxplots by noting where the notches do not overlap which signifies a statistically significant difference. The boxplots for ALL appear to broadly follow those for cannabis use. The applicable Chi-squared test for trend for ALLRs by quintile is significant (Chi Squ. = 613.79, df = 112, *P* = 3.04 × 10^− 70^).

Comparing the highest cannabis use quintile with the remainder 10,326 ALL cases were reported across all ages 2003–2017 in the highest cannabis use states from a total cumulative population of 367,557,212 an overall rate of 2.8091 / 100,000 compared to 60,645 from a cumulative population of 3,509,515,577 in states in lower quintiles an overall rate of 1.7280 / 100,000. These data equate to a risk ratio of 1.6256, a risk difference of 1.0811, an odds ratio (OR) of 1.6256 (95%C.I. 1.52921, 1.6599), an attributable fraction amongst the exposed of 38.4857% (37.6910, 39.2972%), and a population attributable fraction (PAF) of 0.0560 (0.0548, 0.0572), *P* < < 10^− 320^. However this is obviously an underestimate as populations in lower cannabis use quintiles were also exposed to rising rates of cannabinoid exposure overall: that is to say there was a “moving baseline”.

Table 4 lists some key introductory linear regressions of the ALLR against time, cannabis, substances and quintiles. Cannabis use quintiles have also been dichotomized as the upper two quintiles v. the lower three quintiles. In each case significant results are noted. The β-estimate coefficient for ALL regressed against cannabis use alone is 0.2967 (0.1988–0.3945), *P* = 4.25 × 10^− 7^.

**Table 4:** Linear regression models

Parameter	Parameter		Model Parameters				
	Estimate (C.I.)	*P*-Value	SD	R-Squared	F	dF	P
***lm (ALL_Rate ~ Cannabis)***							
Cannabis	0.3 (0.18, 0.41)	4.3E-07	4.06E-01	0.4728	26.26	1508	4.2E-07
***lm (ALL_Rate ~ Time)***							
Year	0.01 (0, 0.01)	0.1670	4.06E-01	0.0018	1.916	1508	0.1670
lm (ALL_Rate ~ Time * Cannabis)							
Year	0.04 (−0.18, −0.02)	0.0130	0.4039	0.05638	11.14	3506	4.3E-07
Cannabis	30.51 (13.22, 132.81)	0.0171					
Year: Cannabis	−0.02 (−0.07, − 0.01)	0.0176					
***Additive model***							
***lm (ALL_Rate ~ Cigarettes + AUD + Cannabis + Analgesics + Cocaine)***							
cigmon	−3.3 (− 4.19, −2.41)	1.3E-12	0.382	0.1561	32.38	3506	< 2.2E-16
AUD	7.58 (5.17, 9.99)	1.5E-09					
Cannabis	0.14 (0.03, 0.26)	0.0154					
***Quintiles***							
***lm (ALL_Rate ~ Quintile)***							
Quintile 4	0.18 (0.07, 0.29)	0.0020	0.4071	0.0234	4.047	4505	0.0031
Quintile 5	0.19 (0.06, 0.32)	0.0037					
lm (ALL_Rate ~ Year * Quintile)							
Year	0.03 (0.01, 0.05)	0.0061	0.4071	0.0416	3.456	9500	0.0004
Quintile 3	79.2 (26.91, 131.49)	0.0031					
Quintile 4	74.23 (21.02, 127.44)	0.0065					
Quintile 5	61.55 (3.2, 119.9)	0.0392					
Year: Quintile 3	−0.04 (− 0.07, − 0.01)	0.0032					
Year: Quintile 4	− 0.04 (− 0.06, − 0.01)	0.0066					
Year: Quintile 5	− 0.03 (− 0.06, 0)	0.0397					
lm (ALL_Rate ~ Year * Quintiles_Dichotomized)							
Upper Quintiles	0.15 (0.07, 0.22)	0.0001	0.4102	0.0272	15.24	1508	0.0001

Inverse probability weights can be calculated on this data for cannabis exposure as a function of other substance exposure.

Inverse probability weighted mixed effects models can be computed from this data with results shown in Table 5. Cannabis use is shown to be highly and independently significant both alone and in additive models including all substances, income and all ethnicities. Terms including cannabis are also persistently significant in final interactive models.

**Table 5:** Mixed effects regression models

Parameter Estimates			Model Parameters			
Parameter	Estimate (C.I.)	*P*-Value	S.D.	AIC	BIC	logLik
***ADDITIVE MODELS***						
***lme (ALL_Rate ~ Cannabis)***						
Cannabis	0.33 (0.15, 0.51)	0.0004	0.4689	872.1647	889.0866	− 432.0824
***Drugs***						
***lme (ALL_Rate ~ Cigarettes + Cannabis + AUD + Analgesics + Cocaine)***						
Cannabis	0.43 (0.23, 0.62)	2.7E-05	0.4668	863.5448	884.6873	− 426.7724
Cocaine	10.44 (2.01, 18.87)	0.0156				
***Drugs, Income & Ethnicity***						
***lme (ALL_Rate ~ Cigarettes + Cannabis + AUD + Analgesics + Cocaine + Income + 5_Races)***						
Hispanic	0.45 (0.33, 0.57)	2.7E-12	0.4478	813.8363	860.2190	−395.9182
Cigarettes	5.21 (3.52, 6.9)	3.0E-09				
Income	1 (0.56, 1.44)	1.2E-05				
Cannabis	0.25 (0.06, 0.44)	0.0095				
Analgesics	−4.44 (−9.7, 0.82)	0.0986				
Asian-American	−0.14 (−0.29, 0.02)	0.0833				
AIAN-American	−4.72 (−8.06, −1.39)	0.0057				
African-American	−0.1 (− 0.17, − 0.03)	0.0056				
***INTERACTIVE MODEL***						
lme (ALL_Rate ~ Cigarettes * Cannabis * AUD + Analgesics + Cocaine + Income + 5_Races)						
Hispanic	0.37 (0.28, 0.46)	7.0E-15	0.4508	809.6203	851.8063	−394.8101
Cannabis: AUD	7.95 (4.69, 11.22)	2.5E-06				
AUD	20.43 (11.44, 29.43)	1.1E-05				
Income	0.85 (0.47, 1.23)	1.3E-05				
AIAN-American	−4.48 (−7.56, − 1.4)	0.0045				
African-American	−0.12 (−0.19, − 0.06)	0.0003				
Cigarettes: Cannabis	−1.83 (−2.52, − 1.15)	2.6E-07				

Similar results are found at inverse probability weighted panel regression (Table 6). In additive and interactive models cannabis use is independently significant in models including all substances, income and ethnicities (from β-estimate = 5.52 (3.71–7.34), *P* = 4.71 × 10^− 9^). When the cannabinoids cannabigerol and tetrahydrocannabinol (THC) are considered, cannabigerol is significant from β-estimate = 1.21 (0.86, 1.56), *P* = 1.39 × 10^− 11^).

**Table 6:** Panel regression models

Parameters				Model				
**Instrumental Variables**	**Parameter**	**Estimate (C.I.)**	***P***-Value	**Adj. R-Squared**	**Chi Squared**	**F**	**dF**	**P**
	***Additive Model***							
	***plm (ALL_Rate ~ Cigarettes + Cannabis + AUD + Analgesics + Cocaine + Income + 5_Races)***							
	Hispanic	0.2 (0.12, 0.27)	5.7E-07	0.1337		−55.3956	6503	1.0000
	Cannabis	−0.22 (−0.33, − 0.11)	1.0E-04					
	Cigarettes	−1.76 (−2.56, −0.96)	2.0E-05					
	Analgesics	−12.01 (− 16.6, − 7.43)	4.0E-07					
	Black	−0.13 (− 0.18, − 0.09)	3.7E-08					
	AUD	−8.02 (−8.89, −7.15)	< 2.2E-16					
	***Interactive Model***							
	plm (ALL_Rate ~ Cigarettes * Cannabis * AUD + Analgesics + Cocaine + Income + 5_Races)							
	Cigarettes: AUD	1157.78 (876.06, 1439.5)	5.9E-15	0.0848		−29.2033	10,499	1.0000
	AIAN	5.35 (3.79, 6.9)	4.4E-11					
	Cigarettes: Cannabis: AUD	416.5 (291.07, 541.94)	1.9E-10					
	Cannabis	5.52 (3.71, 7.34)	4.7E-09					
	Cocaine	10.32 (3.78, 16.86)	0.0021					
	Analgesics	−7.98 (−12.56, −3.41)	0.0007					
	Cannabis: AUD	−87.24 (−115.7, −58.77)	3.7E-09					
	Cigarettes: Cannabis	−28.11 (−36.54, −19.68)	1.6E-10					
	AUD	− 236.82 (− 295.84, − 177.8)	2.3E-14					
	Cigarettes	−83.55 (−103.35, −63.75)	1.2E-15					
	***Interactive Cannabinoids***							
	plm (ALL_Rate ~ Cigarettes + THC * Cannabigerol + AUD + Analgesics + Cocaine + Income + 5_Races)							
	Cannabigerol	1.21 (0.86, 1.56)	1.4E-11	0.0359	−320.949		8	1
	Cocaine	6.84 (3.91, 9.78)	5.0E-06					
	Hispanic	0.18 (0.08, 0.28)	0.0003					
	Asian	−0.13 (− 0.26, − 0.01)	0.0392					
	Analgesics	−13.67 (− 18.79, −8.54)	1.7E-07					
	THC: Cannabigerol	−0.61 (− 0.73, − 0.49)	< 2.2E-16					
	Cigarettes	−4.89 (−5.71, − 4.08)	< 2.2E-16					
	THC	−3.21 (−3.73, −2.69)	< 2.2E-16					
	***Additive Model***							
	***plm (ALL_Rate ~ Cigarettes + Cannabis + AUD + Analgesics + Cocaine + Income + 5_Races)***							
Cauc-Am._THC Exposure	Hispanic-American	0.18 (0.13, 0.23)	2.6E-11	0.3279	254.327		6	2.6E-11
Afric-Am._THC Exposure	Asian-American	0.1 (0.02, 0.18)	0.0126					
Hispan-Am._THC Exposure	Cannabis	0.12 (0, 0.23)	0.0462					
Asian-Am._THC Exposure	Analgesics	−3.72 (−7.32, −0.12)	0.0431					
	Income	− 0.42 (− 0.7, − 0.14)	0.0037					
	African-American	−0.13 (− 0.17, − 0.09)	1.7E-12					

However when ethnic exposure to THC is included in panel models as instrumental variables, the significance of the cannabis effect is greatly reduced (β-estimate = 0.117 (0.002, 0.232), *P* = 0.0462).

Table 7 extends these panel regression results by presenting the results of models lagged to two, four, six and eight years. Highly significant results for terms including cannabis are noted at each time-lag.

**Table 7:** Lagged panel regression models

Parameters				Model			
**Lagged Variables**	**Parameter**	**Estimate (C.I.)**	***P***-Value	**Adj. R-Squared**	**F**	**dF**	**P**
	***Interactive Model 2 Lags***						
	plm (ALL_Rate ~ Cigarettes * Cannabis * AUD + Analgesics + Cocaine + Income + 5_Races)						
Cigarettes, 2	AUD	252.85 (183.13, 322.57)	4.94E-12	0.1084	− 21.106	12,429	1
AUD, 2	Cannabis: AUD	98.16 (65.56, 130.76)	7.31E-09				
Cannabis, 2	Cigarettes	60.32 (36.61, 84.04)	9.01E-07				
Analgesics, 2	Cigarettes: Cannabis	24.47 (14.69, 34.25)	1.34E-06				
Cocaine, 2	Income	1.01 (0.57, 1.44)	6.69E-06				
	Analgesics	10.02 (5.38, 14.65)	2.81E-05				
	Cigarettes: Cannabis: AUD	− 372.02 (−515.25, − 228.79)	5.35E-07				
	African-American	−0.17 (− 0.24, − 0.11)	1.62E-07				
	Cigarettes: AUD	−933.3 (− 1263.65, − 602.95)	5.36E-08				
	Cocaine	−18.93 (−25.45, − 12.41)	2.39E-08				
	Cannabis	−6.95 (− 9.04, −4.86)	2.07E-10				
	White	−1.7 (−2.15, − 1.24)	9.93E-13				
	***Interactive Model 4 Lags***						
	plm (ALL_Rate ~ Cigarettes * Cannabis * AUD + Analgesics + Cocaine + Income + 5_Races)						
Cigarettes, 4	Cigarettes: Cannabis: AUD	1080.24 (773.54, 1386.94)	2.3E-11	0.0348	−24.7061	10,363	1
AUD, 4	Cigarettes: AUD	2872.8 (2029.96, 3715.63)	9.0E-11				
Cannabis, 4	Cannabis	18.57 (12.96, 24.18)	2.9E-10				
Analgesics, 4	Hispanic	0.27 (0.15, 0.39)	1.1E-05				
Cocaine, 4	AIAN-American	−2.93 (−5.5, − 0.36)	0.0258				
	African-American	−0.14 (− 0.21, − 0.07)	5.8E-05				
	Cigarettes	−168.92 (−237.17, −100.67)	1.8E-06				
	Cigarettes: Cannabis	−65.26 (−89.6, −40.91)	2.5E-07				
	AUD	− 786.09 (−978.02, −594.16)	1.4E-14				
	Cannabis: AUD	−291.97 (−363.15, − 220.8)	1.3E-14				
	***Interactive Model 6 Lags***						
	plm (ALL_Rate ~ Cigarettes * Cannabis * AUD + Analgesics + Cocaine + Income + 5_Races)						
Cigarettes, 6	Cannabis: AUD	334.49 (201.76, 467.22)	1.3E-06	0.0748	−19.0948	12,293	1
AUD, 6	AUD	854.26 (489.45, 1219.07)	6.6E-06				
Cannabis, 6	Asian	0.33 (0.18, 0.47)	2.8E-05				
Analgesics, 6	Cigarettes: Cannabis	84.43 (40.2, 128.65)	0.0002				
Cocaine, 6	Cigarettes	216.5 (90.79, 342.21)	0.0008				
	Analgesics	7.94 (1.08, 14.8)	0.0241				
	Cigarettes: AUD	− 3188.8 (− 4708.15, − 1669.45)	5.1E-05				
	Cannabis	−23.42 (−34.15, − 12.7)	2.5E-05				
	Cigarettes: Cannabis: AUD	− 1262.2 (− 1805.43, −718.97)	7.7E-06				
	Cocaine	−22.47 (−31.69, − 13.25)	2.8E-06				
	African-American	−0.31 (− 0.41, − 0.2)	5.6E-08				
	White	−2.14 (− 2.71, −1.58)	1.1E-12				
	***Interactive Model 8 Lags***						
	plm (ALL_Rate ~ Cigarettes * Cannabis * AUD + Analgesics + Cocaine + Income + 5_Races)						
Cigarettes, 8	Cigarettes: Cannabis: AUD	599.85 (483.48, 716.22)	< 2.2E-16	− 0.0014	−21.9419	92,228	1
AUD, 8	Cigarettes: AUD	923.42 (657.01, 1189.82)	9.4E-11				
Cannabis, 8	Cigarettes	59.16 (38.99, 79.32)	2.9E-08				
Analgesics, 8	AIAN-American	9.11 (5.26, 12.95)	5.8E-06				
Cocaine, 8	Asian	0.36 (0.17, 0.54)	0.0002				
	African-American	−0.21 (− 0.33, − 0.08)	0.0011				
	Analgesics	−19.12 (−27.41, − 10.82)	1.0E-05				
	AUD	−225.61 (− 289.34, − 161.88)	4.1E-11				
	Cannabis: AUD	−142.95 (− 170.37, − 115.54)	< 2.2E-16				

Robust regression in inverse probability weighted marginal structural models was conducted on this data with results shown in Table 8. Interactive models for all substances, income and five ethnicities are shown. In both cases cannabis is significant both independently and in interaction with other substances.

**Table 8:** Robust regression models

Parameter	Estimate (C.I.)	*P*-Value
***Interactive Model - Drugs***		
svyglm (ALL_Rate ~ Cigarettes * Cannabis * AUD + Analgesics + Cocaine)		
Cigarettes: AUD	735.98 (212.93, 1259.02)	0.0110
Cigarettes: Cannabis: AUD	275.98 (78.23, 473.74)	0.0115
Cannabis	3.42 (0.52, 6.31)	0.0297
Cannabis: AUD	−65.86 (−107.03, −24.69)	0.0045
AUD	−168.05 (− 261.96, −74.14)	0.0018
***Interactive Model - Drugs, Income, Ethnicity***		
svyglm (ALL_Rate ~ Cigarettes * Cannabis * AUD + Analgesics + Cocaine + Income + 5_Races)		
AIAN-American	5.61 (3.91, 7.31)	8.8E-07
Cigarettes: AUD	1076.38 (277.07, 1875.68)	0.0141
Cigarettes: Cannabis: AUD	372.8 (70.03, 675.57)	0.0235
Cannabis	4.75 (0.48, 9.02)	0.0389
Cigarettes: Cannabis	−25.81 (− 48.88, − 2.75)	0.0378
Cannabis: AUD	−74.01 (− 133.03, − 15)	0.0213
Cigarettes	−80.66 (−143.67, − 17.64)	0.0190
AUD	− 207.46 (−347.32, −67.6)	0.0075

**Fig. 9:**
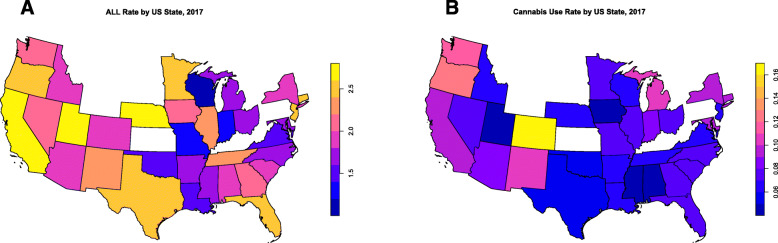
USA States with data for geospatial analysis (**A**) as ALL choropleth map for 2017 and (**B**) Cannabis use choropleth map for 2017

Data on ALLRs by state was complete or almost complete for 34 states. In 14/576 cases missing data was completed by temporal kriging as described above. The states for which data was available are shown in the maps in Fig. 9 which illustrate the 2017 rates of (A) ALL and (B) cannabis use respectively.

**Fig. 10:**
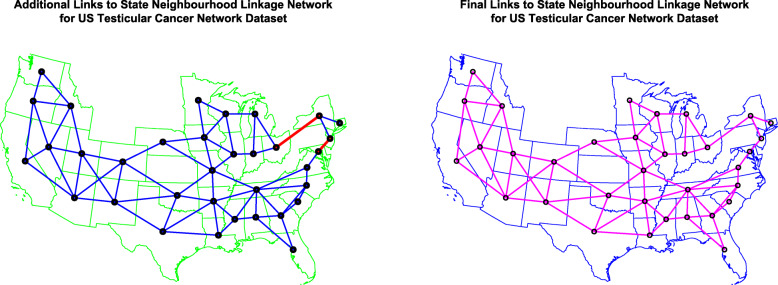
Choropleth Map of state neighbourhood links (**A**) edited and (**B**) final

Figure 10 presents the geospatial neighbourhood links (A) edited after derivation from the spdep::poly2nb function and (B) in final form.

The results of initial spatiotemporal models are shown in Table 9. Cannabis is again independently predictive of ALLR by itself and in additive models with other substances. In a full interactive model with cannabinoids and other substances, income and ethnicities, cannabigerol remains independently significant in the final model.

**Table 9:** Geospatiotemporal regression models

Parameter			Model				
Parameter	Estimate (C.I.)	*P*-Value	LogLik	S.D.	Model Parameter	Estimate	*P*-Value
***Cannabis Alone***							
***spreml (ALL_Rate ~ Cannabis)***							
Cannabis	0.22 (0.09, 0.36)	0.0015	− 193.8962	0.4040	phi	0.5060	0.0005
***Additive Model***							
***spreml (ALL_Rate ~ Cigarettes + Cannabis + AUD + Analgesics + Cocaine)***							
Cannabis	0.16 (0.06, 0.27)	0.0032	N/A	0.3869	phi	0.4604	0.0005
					rho	−0.3274	0.0229
***Interactive Models - Drugs***							
spreml (ALL_Rate ~ Cigarettes * Cannabis * AUD + Analgesics + Cocaine)							
Cigarettes: Cannabis: AUD	22.02 (10.13, 33.91)	0.0003	−189.4824	0.3760	phi	0.3669	0.0022
Cigarettes: AUD	45.76 (17.83, 73.69)	0.0013			rho	−0.3098	0.0349
Cannabis: AUD	−2.39 (−4.75, −0.02)	0.0482			lambda	0.2865	0.0162
***Interactive Models - Drugs, Income, Ethnicity***							
spreml (ALL_Rate ~ Cigarettes * Cannabis * AUD + Analgesics + Cocaine + Income + 5_Races)							
Hispanic	0.21 (0.15, 0.26)	8.3E-13	−170.6536	N/A	rho	0.3314	0.0007
Asian	0.12 (0.04, 0.2)	0.0035			lambda	−0.3523	0.0011
Income	−0.29 (− 0.57, − 0.01)	0.0404					
African-Am.	− 0.16 (− 0.21, − 0.12)	6.2E-14					
***Interactive Cannabinoid Models - Drugs, Income, Ethnicity***							
spreml (ALL_Rate ~ Cigarettes * THC * CBG + AUD + Analgesics + Cocaine + Income + 5_Races)							
Hispanic	0.23 (0.17, 0.28)	9.9E-15	− 163.7442	0.3325	phi	0.0495	0.1403
Cannabigerol	0.26 (0.01, 0.52)	0.0444			psi	0.0573	0.2640
Cigarettes: THC: Cannabigerol	−1.18 (−1.87, −0.49)	0.0008			rho	0.1791	0.2751
Cigarettes: THC	−4.96 (−7.8, −2.12)	0.0006			lambda	−0.2047	0.2159
African-Am.	−0.13 (− 0.18, − 0.09)	3.4E-09					

Technical Notes:

phi: - Idiosyncratic component of the spatial error term

psi: - Individual time-invariant component of the spatial error term

rho: - Spatial autoregressive parameter

lambda: - Spatial autocorrelation coefficient

As cannabigerol was the most powerful term in these spatial models, lagged models were explored where cannabigerol was lagged spatially and temporally. These results are presented in Table 10. Once again terms including THC and cannabigerol are significant and THC and cannabigerol are both independently significant with positive coefficients.

**Table 10:** Lagged geospatiotemporal regression models

Lagged Variables	Parameter			Model				
	Parameter	Estimate (C.I.)	*P*-Value	LogLik	S.D.	Model Parameter	Estimate	*P*-Value
	***Spatial Lagging - 1 Spatial Lag***							
	spreml (ALL_Rate ~ Cigarettes * THC* Cannabigerol + AUD + Analgesics + Cocaine + Income + 5_Races)							
CBG, 1	Hispanic	0.19 (0.14, 0.25)	3.8E-11	− 161.6159	N/A	rho	0.3329	0.0005
	Asian	0.15 (0.07, 0.23)	0.0003			lambda	−0.3858	0.0002
	THC	0.47 (0.12, 0.82)	0.0083					
	Income	−0.43 (−0.72, − 0.13)	0.0045					
	Cigarettes: THC: Cannabigerol	− 2.67 (− 3.97, − 1.37)	5.9E-05					
	Cigarettes: THC	−11.45 (− 16.99, − 5.9)	5.2E-05					
	African-Am.	− 0.18 (− 0.22, − 0.14)	2.3E-16					
	***Spatial Lagging - 2 Spatial Lags***							
	spreml (ALL_Rate ~ Cigarettes * THC* Cannabigerol + AUD + Analgesics + Cocaine + Income + 5_Races)							
CBG, 2	Hispanic	0.21 (0.15, 0.27)	3.0E-12	−162.0804	N/A	rho	0.2875	0.0065
	Asian	0.14 (0.06, 0.22)	0.0006			lambda	− 0.3379	0.0024
	Cannabigerol	0.80 (0.16, 1.43)	0.0140					
	Cigarettes	−10.92 (−21.53, −0.3)	0.0439					
	Cigarettes: Cannabigerol	− 3.42 (−6.41, − 0.42)	0.0253					
	Income	−0.37 (− 0.69, − 0.05)	0.0252					
	Cigarettes: THC	−8.23 (−13.46, − 3.01)	0.0020					
	Cigarettes: THC: Cannabigerol	− 2.35 (− 3.82, − 0.89)	0.0016					
	African-Am.	− 0.17 (− 0.21, − 0.13)	7.3E-15					
	***Temporal Lagging - 1 Lag***							
	spreml (ALL_Rate ~ Cigarettes * THC* Cannabigerol + AUD + Analgesics + Cocaine + Income + 5_Races)							
CBG, 1	Hispanic	0.22 (0.15, 0.29)	1.1E-09	− 139.8349	N/A	psi	0.0981	0.0430
	Cannabigerol	0.82 (0.25, 1.39)	0.0046			rho	0.1312	0.4054
	Asian	0.12 (0.03, 0.2)	0.0093			lambda	−0.2103	0.1734
	AIAN	−2.2 (−4.08, −0.32)	0.0217					
	Cigarettes	−12.59 (−22.78, − 2.39)	0.0155					
	Cigarettes: Cannabigerol	−3.73 (−6.58, −0.89)	0.0101					
	Income	−0.46 (− 0.81, − 0.11)	0.0099					
	Cigarettes: THC	−6.13 (−9.34, − 2.93)	0.0002					
	Cigarettes: THC: Cannabigerol	−1.73 (− 2.61, − 0.86)	9.7E-05					
	African-Am.	−0.2 (− 0.24, − 0.15)	< 2.2E-16					
	***Temporal Lagging - 2 Lags***							
	spreml (ALL_Rate ~ Cigarettes * THC* Cannabigerol + AUD + Analgesics + Cocaine + Income + 5_Races)							
CBG, 2	Hispanic	0.18 (0.12, 0.24)	2.0E-09	−132.1090	N/A	rho	0.2545	0.0368
	Asian	0.16 (0.07, 0.24)	0.0003			lambda	−0.2987	0.0169
	Cigarettes: THC	−3.05 (−5.82, −0.28)	0.0308					
	Cigarettes: THC: Cannabigerol	−0.86 (−1.61, − 0.12)	0.0226					
	Income	−0.51 (− 0.81, − 0.21)	0.0010					
	African-Am.	− 0.18 (− 0.22, − 0.14)	6.9E-16					

Technical Notes:

phi: - Idiosyncratic component of the spatial error term

psi: - Individual time-invariant component of the spatial error term

rho: - Spatial autoregressive parameter

lambda: - Spatial autocorrelation coefficient

Table 11 explores the effects of ethnic THC exposure in more detail in three models, additive for ethnic THC exposure, interactive for ethnic THC exposure, and interactive for various substances and interactive for ethnic THC exposure together. In all cases ethnic THC exposure is significant with positive coefficients.

**Table 11:** Geospatiotemporal ethnic regression models

Parameter			Model				
Parameter	Estimate (C.I.)	*P*-Value	LogLik	S.D.	Model Parameter	Estimate	*P*-Value
***Ethnic THC Exposure - Additive***							
***spreml (ALL_Rate ~ Caucasian-Am._THC_Exp + African-Am._THC_Exp + Hispanic-Am._THC_Exp + Asian-AM._THC_Exp + AIAN-Am.THC_Exp + NHPI_THC_Exp)***							
HispanicTHC_Expos.	0.39 (0.13, 0.65)	0.0038	−190.5057	0.3912	phi	0.5148	0.0005
NHAfrican-Am.THC_Expos.	0.34 (0.06, 0.63)	0.0184			rho	−0.3454	0.0099
NHCaucas.-Am.THC_Expos.	−0.75 (−1.25, − 0.25)	0.0032			lambda	0.2982	0.0063
***Ethnic THC Exposure - Interactive***							
spreml (ALL_Rate ~ Caucasian-Am._THC_Exp * African-Am._THC_Exp * Hispanic-Am._THC_Exp + Asian-AM._THC_Exp + AIAN-Am.THC_Exp + NHPI_THC_Exp)							
NHCaucas.-Am.THC_Expos.: NHAfrican-Am.THC_Expos.: HispanicTHC_Expos.	0.12 (0.04, 0.2)	0.0031	− 1847.4309	0.3817	phi	0.5033	0.0004
NHCaucas.-Am.THC_Expos.	−0.06 (− 0.11, 0)	0.0349			rho	−0.4053	0.0005
NHCaucas.-Am.THC_Expos.: HispanicTHC_Expos.	−0.1 (− 0.16, − 0.04)	0.0007			lambda	0.3359	0.0003
***Ethnic THC Exposure - Interactive in Substances and in Ethnicity***							
spreml (ALL_Rate ~ Cigarettes * AUD + Analgesics + Cocaine + Caucasian-Am._THC_Exp * African-Am._THC_Exp * Hispanic-Am._THC_Exp + Asian-AM._THC_Exp + AIAN-Am.THC_Exp + NHPI_THC_Exp)							
NHCaucas.-Am.THC_Expos.: NHAfrican-Am.THC_Expos.: HispanicTHC_Expos.	0.12 (0.04, 0.2)	0.0023	−180.6319	0.3642	phi	0.3436	0.0021
Cigarettes: AUD	16.13 (3.39, 28.87)	0.0131			rho	−0.3769	0.0016
NHCaucas.-Am.THC_Expos.	−0.08 (− 0.16, − 0.01)	0.0305			lambda	0.3057	0.0014
Cigarettes	− 2.86 (− 4.67, −1.05)	0.0019					
NHCaucas.-Am.THC_Expos.: HispanicTHC_Expos.	−0.12 (− 0.18, − 0.06)	7.5E-05					

Technical Notes:

phi: - Idiosyncratic component of the spatial error term

psi: - Individual time-invariant component of the spatial error term

rho: - Spatial autoregressive parameter

lambda: - Spatial autocorrelation coefficient

**Fig. 11:**
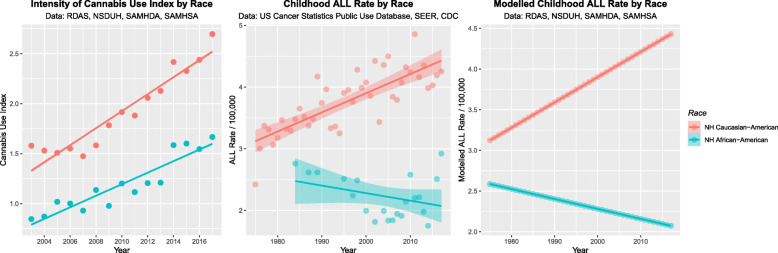
(**A**) Cannabis use intensity for Caucasian -Americans and African-Americans; (**B**) Childhood ALL Rates by Race for both ethnicities and (**C**) modelled ALL rates over the whole time period by ethnicity

Figure 11 (A) shows the intensity of last month cannabis use for Caucasian-American and African-American ethnicities. Fig. 11 (B) shows the SEER dataset for the ALLRs by these two ethnicities. Fig. 11 (C) projects this data out over the whole time period 1975–2017 based on extensions of the linear models. The mean state ethnicity ALLRs are 3.47 ± 0.13 and 2.47 ± 0.27 (mean ± S.E.M., /100,000) for Caucasian-American and African-Americans respectively (t = 24.03, df = 101.72, *P* = 4.91 × 10^− 44^)

These interesting and provocative ethnic differences between the Caucasian-American and the African-American populations invited further exploration. However 489 of the of the 1020 (47.94%) datapoints were missing or suppressed. These were imputed by the multiple imputation by chained equations routine in R package mice. Following [46, 47] 60 imputations each with 60 iterations were employed due to the large amount of missing data.

**Fig. 12:**
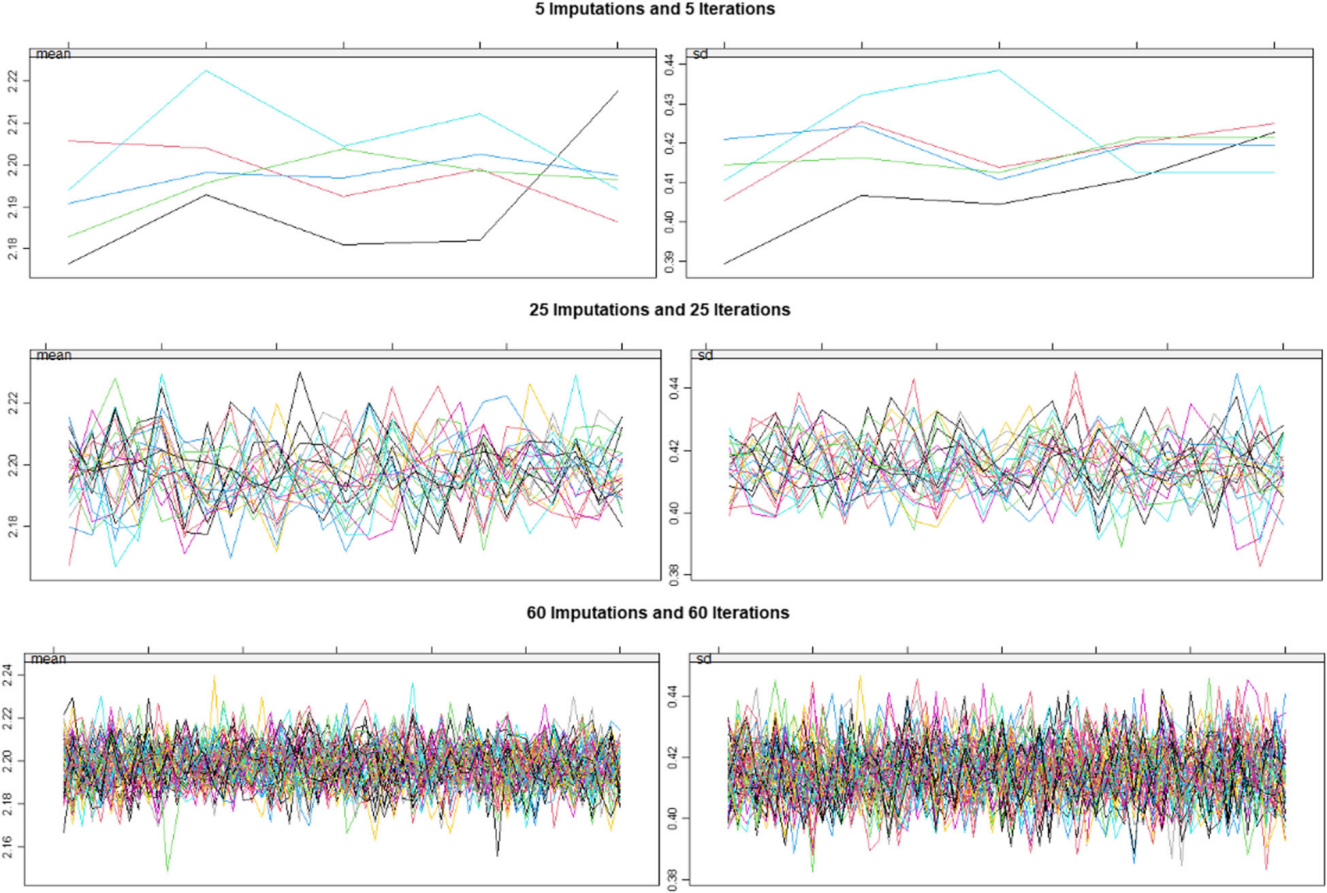
Stripplots showing convergence of the multiple imputation algorithm with increasing numbers of imputations and iterations to 5 and 5, 25 and 25 and 60 and 60 respectively

Figure 12 shows successful convergence of the imputations as stripplots with successively increasing imputations and iterations.

**Fig. 13:**
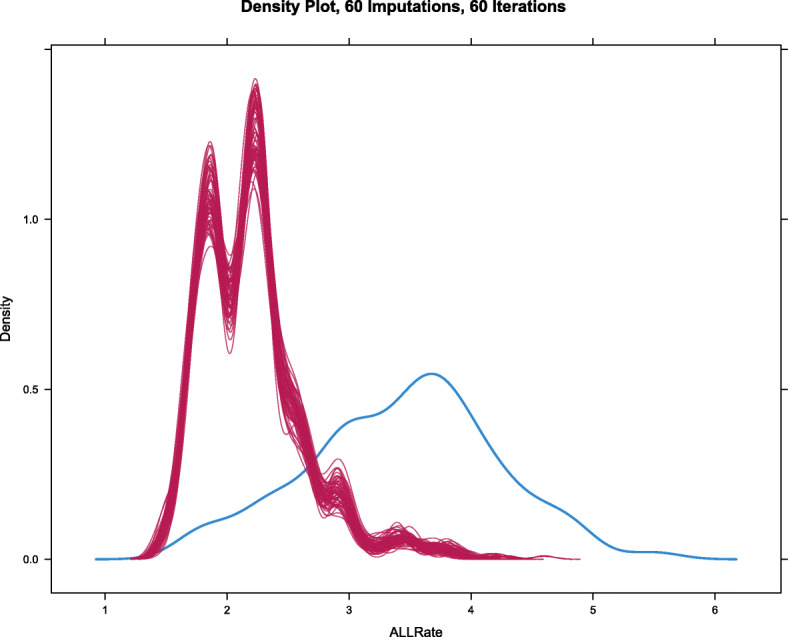
Density plot of imputed values in the various imputations

Figure 13 shows the density plot of the imputed data. Imputed data are shifted relative to the main dataset as the imputations occurred primarily in the ethnic minorities which had a lower mean ALLR.

Table 12 presents the results of linear regression on the imputed datasets. The ALLR is noted to be highly significantly related to ethnic THC exposure alone (β-estimate = 0.14 (0.12, 0.17), *P* = 3.4 × 10^− 26^), and ethnic THC exposure is independently highly predictive in an additive (β-estimate = 0.53 (0.44, 0.61), *P* = 6.2 × 10^− 31^) and an interactive (β-estimate = 0.42 (0.35, 0.50), *P* = 1.7 × 10^− 27^) model. When cannabinoids are included as primary covariates, ethnic THC exposure remains significant in interactive terms. Indeed ethnic THC exposure remains significant in this table as model complexity increases.

**Table 12:** Linear models from imputed data

Parameters			Model			
Parameter	Estimate (C.I.)	*P*-Value	No. Imputa- tions	SD	lambda	FMI
***Cannabis Alone***						
***lm (ALL_Rate ~ Ethnic_THC_Exposure***						
Ethnic_THC_Exposure	0.14 (0.12, 0.17)	3.4E-26	60	0.2891	0.0562	0.0583
***Additive Model***						
***lm (ALLRate ~ Cigarettes + AUD + Ethic_THC_Exposure + Analgesics + Cocaine)***						
Ethnic_THC_Exposure	0.53 (0.44, 0.61)	6.2E-31	60	0.8246	0.0301	0.0321
AUD	9.1 (5.3, 12.89)	3.1E-06			0.0301	0.0321
Cigarettes	1.52 (0.09, 2.95)	0.0371			0.0294	0.0314
***Interactive Model***						
***lm (ALLRate ~ Cigarettes + AUD + Ethic_THC_Exposure + Analgesics + Cocaine + Cigarettes: AUD + Cigarettes: Ethnic_Cannabis + AUD: Ethnic_Cannabis)***						
Ethnic_THC_Exposure	0.42 (0.35, 0.5)	1.7E-27	60	0.8347	0.0335	0.0355
Cocaine	10.69 (2.47, 18.92)	0.0110			0.0323	0.0343
***Interactive Model with Cannabinoids***						
***lm (ALLRate ~ Cigarettes + AUD + THC + Cannabigerol + Analgesics + Cocaine + Cigarettes: AUD + Cigarettes: Ethnic_Cannabis + AUD: Ethnic_Cannabis)***						
Cigarettes: Ethnic_THC_Exposure	3.77 (3.12, 4.42)	5.8E-13	60	0.6885	0.0270	0.0290
Cannabigerol	2.14 (1.65, 2.63)	2.1E-17			0.0484	0.0505
AUD: Ethnic_THC_Exposure	6.98 (5.08, 8.88)	1.2E-12			0.0251	0.0270
Cigarettes	−5.68 (−7.03, −4.33)	4.4E-16			0.0596	0.0617
THC: Cannabigerol	−19.56 (−22.57, − 16.55)	1.0E-34			0.0405	0.0425
THC	−2.63 (−3.03, − 2.23)	1.3E-35			0.0538	0.0559
***Interactive Model with Ethnicity & Income***						
***lm (ALLRate ~ Cigarettes + AUD + Ethic_THC_Exposure + Analgesics + Cocaine + Cigarettes: AUD + Cigarettes: Ethnic_Cannabis + AUD: Ethnic_Cannabis + Income + NHWhite + NHAfrican-Am.)***						
Ethnic_THC_Exposure	0.64 (0.55, 0.72)	3.1E-40	60	0.8028	0.0352	0.0373
Cigarettes: AUD	27.3 (14.38, 40.22)	3.7E-05			0.0328	0.0348
Analgesics	−8.83 (−14.57, − 3.09)	0.0027			0.0268	0.0288
Income	−1.73E-05 (−2.46E-05, − 1.0E-05	3.7E-06			0.0482	0.0502
Black	−1.56 (−2.18, −0.94)	1.1E-06			0.0371	0.0391
White	−1.28 (− 1.72, −0.84)	2.6E-08			0.0313	0.0333
***Interactive Model with Intensity Cannabis Use***						
Cigarettes: Ethnic_THC_Exposure	2.97 (2.61, 3.32)	4.80E-53	60	0.7883	0.0206	0.0226
Cigarettes: AUD	−31.33 (−44.5, −18.17)	3.53E-06			0.0434	0.0455
NHWhite_Cannabis_Intensity	−1.32 (− 1.55, − 1.09)	6.34E-28			0.0420	0.0440
***Interactive Model with Ethnicity & Income with Intensity Cannabis Use***						
***lm (ALLRate ~ Cigarettes + AUD + Ethnic_Intensity_Cannabis_Use_Indices + Analgesics + Cocaine + Cigarettes: AUD + Cigarettes: Ethnic_Cannabis + AUD: Ethnic_Cannabis + Income + NHWhite + NHAfrican-Am.)***						
Cigarettes: Ethnic_THC_Exposure	4.69 (3.77, 5.61)	1.0E-22	60	0.7585	0.0231	0.0251
Cigarettes: AUD	150 (67.09, 232.91)	4.0E-04			0.0479	0.0500
Cocaine	− 11.7 (−21.38, −2.02)	0.0181			0.0447	0.0467
Income	−1.1E-05 (−2.0E-05, −3.0E-06)	0.0097			0.0458	0.0478
AUD: Ethnic_THC_Exposure	−3.51 (−6.14, −0.88)	0.0088			0.0274	0.0294
AUD	−29.6 (−49.98, −9.22)	0.0045			0.0477	0.0497
White	−0.88 (−1.34, − 0.41)	0.0002			0.0441	0.0462
Analgesics	−11.6 (− 17.11, −6.09)	3.8E-05			0.0316	0.0336
Cigarettes	−17 (−22.66, − 11.34)	5.1E-09			0.0394	0.0415
NHWhite_Cannabis_Intensity	−1.58 (− 1.82, − 1.34)	4.2E-35			0.0472	0.0493

Table 13 collates some of the e-Values calculated from the above analyses. The minimum e-Values are listed in descending order in Table 14. 33 / 35 e-Values are > 1.25 which is the cut-off quoted as indicative of causal effects in the literature [49]. The highest minimum e-Value relates to ethnic cannabis exposure differences by ethnicity (3.94 × 10^36^).

**Table 13:** e-Values

Parameter	Estimate (C.I.)	R.R. (C.I.)	E-Values
***Linear Models***			
***lm (ALL_Rate ~ mrjmon)***			
Cannabis	0.3 (0.18, 0.41)	1.94 (1.51, 2.51)	3.30, 2.38
lm (ALL_Rate ~ Time * mrjmon)			
Cannabis	30.51 (13.22, 132.81)	2.70E+ 71 (1.13E+ 13, 6.46E+ 129)	5.40E+ 71, 2.25E+ 13
***Quintiles***			
***lm (ALL_Rate ~ Quintile)***			
Quintile 4	0.18 (0.07, 0.29)	1.49 (1.15, 1.91)	2.34, 1.59
Quintile 5	0.19 (0.06, 0.32)	1.53 (1.15, 2.04)	2.43, 1.57
lm (ALL_Rate ~ Year * Quintile)			
Quintile 3	79.2 (26.91, 131.49)	7.55E+ 76 (1.67E+ 26, 3.41E+ 127)	1.51E+ 77, 3.34E+ 26
Quintile 4	74.23 (21.02, 127.44)	1.13E+ 72 (3.23E+ 20, 3.96E+ 123)	2.26E+ 72, 6.50E+ 20
Quintile 5	61.55 (3.2, 119.9)	5.54E+ 59 (1.65E+ 03, 1.85E+ 116)	1.11E+ 60, 3.31E+ 03
lm (ALL_Rate ~ Year * Dichotomized_Quintiles)			
Upper Quintiles	0.15 (0.07, 0.22)	1.99E+ 59 (1.56E+ 03, 2.54E+ 115)	3.99E+ 59, 3.13E+ 03
***Legal Status***			
***lm (Rate ~ Status)***			
Legal	0.3 (0.12, 0.49)	1.98 (1.31, 2.98)	3.37, 1.95
Medical	0.21 (0.12, 0.31)	1.62 (1.30, 2.00)	2.62, 1.94
lm (Rate ~ Year * Status)			
Legal	0.3 (0.12, 0.49)	1.98 (1.31, 2.98)	3.37, 1.95
Medical	0.21 (0.12, 0.31)	1.62 (1.30, 2.00)	2.62, 1.94
lm (Rate ~ Year * Dichotomized Status)			
Liberal	0.11 (0.04, 0.18)	1.27 (1.08, 1.49)	1.85, 1.38
***Additive model***			
***lm (ALL_Rate ~ Cigarettes + AUD + Cannabis + Analgesics + Cocaine)***			
Cannabis	0.14 (0.03, 0.26)	1.40 (1.06, 1.84)	2.16, 1.34
***MIXED EFFECTS MODELS***			
***ADDITIVE MODELS***			
***lme (ALL_Rate ~ Cannabis)***			
Cannabis	0.33 (0.15, 0.51)	1.30 (1.04, 2.47)	2.58, 1.24
***Drugs***			
***lme (Rate ~ Cigarettes + Cannabis + AUD + Analgesics + Cocaine)***			
Cannabis	0.43 (0.23, 0.62)	2.29 (1.56, 3.37)	4.02, 2.50
***Drugs, Income & Ethnicity***			
***lme (ALL_Rate ~ Cigarettes + Cannabis + AUD + Analgesics + Cocaine + Income + 5_Races)***			
Cannabis	0.25 (0.06, 0.44)	1.66 (1.14, 2.44)	2.72, 1.53
***INTERACTIVE MODEL***			
lme (ALL_Rate ~ Cigarettes * Cannabis * AUD + Analgesics + Cocaine + Income + 5_Races)			
Cannabis: AUD	7.95 (4.69, 11.22)	9.40E+ 06 (1.29E+ 04, 6.81E+ 09)	1.88E+ 07, 2.59E+ 04
***GEOSPATIAL MODELS***			
***Cannabis Alone***			
***spreml (ALL_Rate ~ Cannabis)***			
Cannabis	0.22 (0.09, 0.36)	3.44 (1.61, 7.36)	6.34, 2.60
***Additive Model***			
***spreml (ALL_Rate ~ Cigarettes + Cannabis + AUD + Analgesics + Cocaine)***			
Cannabis	0.16 (0.06, 0.27)	1.47 (1.14, 1.90)	2.31, 1.54
***Interactive Models - Drugs***			
spreml (ALL_Rate ~ Cigarettes * Cannabis * AUD + Analgesics + Cocaine)			
Cigarettes: Cannabis: AUD	22.02 (10.13, 33.91)	1.38E+ 23 (4.65E+ 10, 4.11E+ 35)	2.77E+ 23, 9.30E+ 10
***Interactive Cannabinoid Models - Drugs, Income, Ethnicity***			
spreml (ALL_Rate ~ Cigarettes * Cannabis * AUD + Analgesics + Cocaine + Income + 5_Races)			
Cannabigerol	0.26 (−3.68, 4.2)	2.04 (1.02, 4.11)	3.51, 1.16
***ETHNICITY MODELS***			
***Ethnic THC Exposure - Additive***			
***spreml (ALL_Rate ~ Caucasian-Am._THC_Exp + African-Am._THC_Exp + Hispanic-Am._THC_Exp + Asian-AM._THC_Exp + AIAN-Am.THC_Exp + NHPI_THC_Exp)***			
HispanicTHC_Expos.	0.39 (0.13, 0.65)	2.47 (1.34, 4.54)	4.37, 2.01
NHAfrican-Am.THC_Expos.	0.34 (0.06, 0.63)	2.11 (1.09, 4.07)	3.65, 1.43
***Ethnic THC Exposure - Interactive***			
spreml (ALL_Rate ~ Caucasian-Am._THC_Exp * African-Am._THC_Exp * Hispanic-Am._THC_Exp + Asian-AM._THC_Exp + AIAN-Am.THC_Exp + NHPI_THC_Exp)			
NHCaucas.-Am.THC_Expos.: NHAfrican-Am.THC_Expos.: HispanicTHC_Expos.	0.12 (0.04, 0.2)	1.33 (1.10, 1.59)	1.98, 1.43
spreml (ALL_Rate ~ Cigarettes * AUD + Analgesics + Cocaine + Caucasian-Am._THC_Exp * African-Am._THC_Exp * Hispanic-Am._THC_Exp + Asian-AM._THC_Exp + AIAN-Am.THC_Exp + NHPI_THC_Exp)			
NHCaucas.-Am.THC_Expos.: NHAfrican-Am.THC_Expos.: HispanicTHC_Expos.	0.12 (0.04, 0.2)	1.36 (1.12, 1.66)	2.06, 1.48
***Ethnic Imputed Models***			
***Additive Model***			
Ethnic_THC_Exposure	0.53 (0.44, 0.61)	1.78 (1.62, 1.96)	2.97, 2.64
***Interactive Model***			
Ethnic_THC_Exposure	0.42 (0.35, 0.5)	1.59 (1.46, 1.72)	2.55, 2.29
***Interactive Model with Cannabinoids***			
Cannabigerol	2.14 (1.65, 2.63)	16.99 (8.90, 34.42)	33.47, 17.29
Cigarettes: Ethic_THC_Exposure	3.77 (3.12, 4.42)	146.54 (62.25, 344.97)	292.59, 124.00
AUD: Ethnic_THC_Exposure	6.98 (5.08, 8.88)	1.01E+ 04 (829.80, 1.24E+ 05)	2.03E+ 04, 1.66E+ 03
***Interactive Model with Race & Income***			
Ethic_THC_Exposure	0.64 (0.55, 0.72)	2.05 (1.85, 2.27)	3.52, 3.11
***Interactive Model with Race & Income & Cannabis Intensity***			
Cigarettes: Ethic_THC_Exposure	4.69 (3.77, 5.61)	279.13 (92.59, 841.47)	557.76, 184.68
***Cannabis_Use_Intensity***			
NH Caucasian-American	8.93 (6.2, 11.66)	1.66E+ 52 (1.97E+ 36, 1.39E+ 68)	3.32E+ 52, 32.94E+ 36

**Table 14:** List of Minimum e-Values

No.	Minimum e-Value
1	3.94E+ 36
2	3.34E+ 26
3	6.50E+ 20
4	2.25E+ 13
5	9.30E+ 10
6	2.54E+ 08
7	25,900.00
8	3310.00
9	3130.00
10	1660.00
11	184.68
12	124.00
13	17.29
14	3.11
15	2.64
16	2.6
17	2.5
18	2.38
19	2.29
20	2.01
21	1.95
22	1.95
23	1.94
24	1.94
25	1.59
26	1.57
27	1.54
28	1.53
29	1.48
30	1.43
31	1.43
32	1.38
33	1.34
34	1.24
35	1.16

**Fig. 14:**
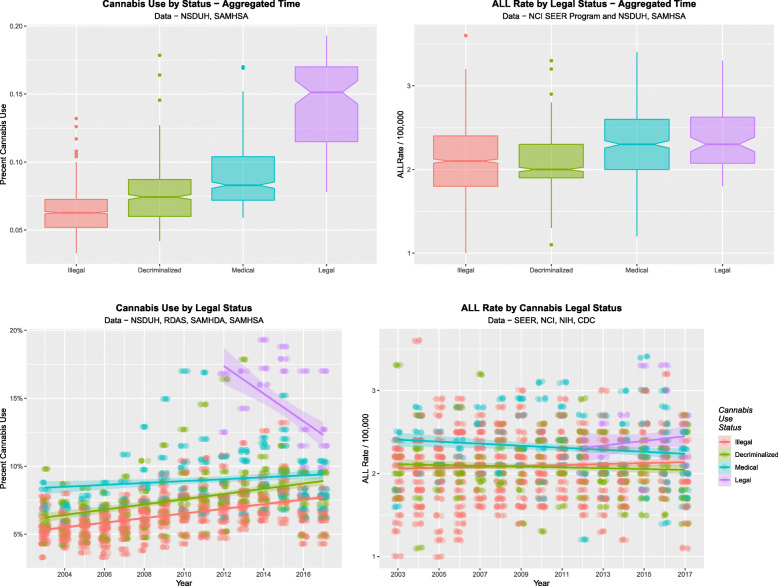
Effect of cannabis legal status on ALL rate. (**A** and **C**) Cannabis use by legal status and (**B** and **D**) ALL rate by legal status as (**C** and **D**) scatterplots and (**A** and **B**) boxplots. Note particularly non-overlapping notches in the boxplots which signify statistically significant differences

Having demonstrated prominent dose-response and quintile effects, the effects of cannabis legalization remain to be considered. Cannabis legalization is associated with higher rates of use, higher intensity of use and higher concentration of THC in cannabis products [50]. The rates of cannabis use (A, C) and ALL (B, D) by legal status are shown in Fig. 14 both as scatterplots (C, D) and boxplots (A, B). The scatterplots and boxplots for ALL appear to track those for cannabis use. The mean ALLR under illegal, decriminalized, medical and legal paradigms were 2.091 ± 0.009, 2.077 ± 0.014, 2.305 ± 0.018 and 2.395 ± 0.039 / 100,000 (mean ± S.E.M.) respectively. The Chi squared test for trend is highly significant (Chi Squ. = 775.12, df = 84, *P* = 2.14 × 10^− 112^).

**Fig. 15:**
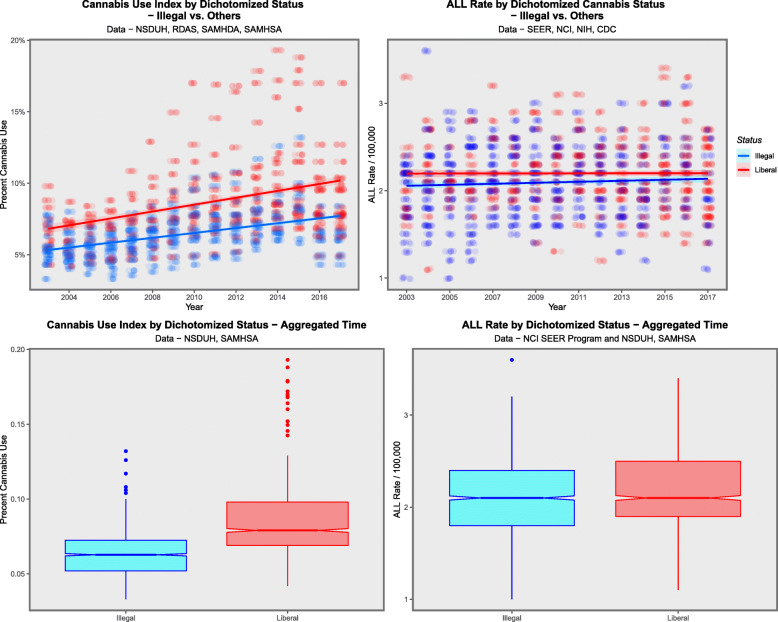
Effect of dichotomized cannabis legal status on ALL rate. Dichotomized as legal status v. not legal. (**A** and **C)** Cannabis use by legal status and (**B** and **D**) ALL rate by legal status as (**C** and **D**) boxplots and (**A** and **B**) scatterplots. Note particularly non-overlapping notches in the boxplots which signify statistically significant differences

Data may be dichotomized as the legal paradigm v. the others as shown in Fig. 15. The notches of the ALL boxplots in the two groups are noted to clearly not overlap. The mean ALLR in the legal and not-legal groups were 2.395 ± 0.039 and 2.127 ± 0.008 / 100,000 respectively (t = 6.7151, df = 128.16, *P* = 5.05 × 10^− 10^).

When these data are analyzed by linear regression the highly significant results shown in Table 15 are found. These results are associated with minimum e-Values mostly > 1.90 as shown in the upper part of Table 13. 33/35 e-Values are > 1.25 which is the cut-off point described in the literature for causal effects 1.25 [49]. 12 e-Values are > 100.

**Table 15:** Legal Status

Parameter	Parameter		Model Parameters				
	Estimate (C.I.)	P-Value	SD	R-Squared	F	dF	P
***Legal Status***							
***lm (Rate ~ Status)***							
Legal	0.3 (0.12, 0.49)	0.0013	0.4053	0.0502	9.971	3506	2.10E-06
Medical	0.21 (0.12, 0.31)	1.4E-05					
lm (Rate ~ Year * Status)							
Legal	0.3 (0.12, 0.49)	0.0013	0.4053	0.0502	9.971	3506	2.10E-06
Medical	0.21 (0.12, 0.31)	1.4E-05					
lm (Rate ~ Year * Dichotomized Status)							
Liberal	0.11 (0.04, 0.18)	0.0037	0.4128	0.0146	8.523	1508	0.0036
lm (Rate ~ Year * Dichotomized Status)							
Legal (v Not Legal)	1.24E-04 (3.0E-05, 2.2E-04)	0.0103	0.4132	0.0127	4.288	2507	0.0142

## Discussion

### Main results

This study significantly extends prior cohort analyses linking cannabis use with the incidence of childhood leukaemia. We here report a positive relationship between cannabis exposure and ALLR for the first time. Importantly data analysis shows that this result is not limited to a single cohort analysis, but is generalizable across the pediatric cancer epidemiology of a whole nation for the most common malignant disease of childhood. Further data indicate that ethnic differences in ALL incidence are associated in part with differing intensity of cannabis use, suggesting a gene-environment interaction. The present report includes other substances, median household income and ethnicity and finds that the effects of exposure to cannabis and cannabinoids is independently significant and persistent in final robust regression models. In bivariate analysis the ALLR was related to AUD, cannabis use and estimates of state-level cannabinoid exposure to THC, cannabinol, cannabigerol, cannabichromene and cannabidiol and strong dose-response effects were demonstrated. Similarly the effect of ethnic THC exposure is maintained across most ethnicities. Inclusion of ethnic THC exposure as either a primary covariate or an instrumental variable greatly mollifies the effect of cannabis exposure per se. The cannabis-ALL link was strongly maintained when analyzed across space and time. The causal nature of the relationship was demonstrated by significant results upon inclusion of inverse probability weights in mixed effects, panel, and robust regression models, and by the demonstration that 33/35 minimum e-Values were above the cut-off threshold of 1.25 extending up to 3.94 × 10^36^. Consistent with the general dose-response effects prominent quintile effects were demonstrated, as were major effects of cannabis legalization which has been linked with increased cannabis availability, use, intensity of use and THC potency [50].

The present study reports a strong, robust, spatiotemporal and causal link between cannabis use and ALLR. Current findings contrast with cohort and case-control studies undertaken two to three decades ago investigating the association between ALL and cannabis where no apparent association was identified [8–10]. However, numerous in vitro studies investigating genotoxic changes associated with cannabinoid exposure have reported that a threshold exposure is required before detrimental changes occur. It is therefore possible that the apparent disparity in findings are explained by increasing frequency of cannabis use, especially amongst existing users, and increased concentration in cannabis products of THC and many other genotoxic cannabinoids over the last two decades [24–26] resulting in a general movement of the whole population into a much higher risk category.

The potential impact of this investigation is far reaching given the possibility that cannabis may be a primary driver for the remarkable 42% rise in total pediatric cancer across the USA from 1975 to 2017 [5]. This relates to the general genotoxicity of cannabis and cannabinoids, to the multiplicity of mechanisms by which cannabinoids act genotoxically and / or epigenotoxically and its evident disruption of chromosomal and epigenomic physiology and to the transgenerational transmission of the effects of environmental intoxicants and thereby the multigenerational impacts of widespread and increasing cannabinoid exposure as is implicit in cannabis legalization paradigms.

### Cellular and biological mechanisms

#### Genetic and chromosomal pathways

Significant data indicate that interchromosomal translocations or gene amplifications can upregulate oncogenes or downregulate tumour suppressors. The classically documented action of cannabinoids including tetrahydrocannabinol (THC), cannabidiol and cannabinol to induce end-to-end chromosomal fusions, ring chromosomes and chain chromosomes in sperm [19], and to cause nuclear blebbing and chromosomal bridges between separating nuclei in anaphase and telophase in dividing oocytes and lymphocytes [51–53] constitute in vitro proof of principal that cannabis is at least an indirect chromosomal clastogen as described long ago [19, 54–60]. Genetic lesions underlying ALL have been proposed by several authors [7, 61–64].

The cannabinoids Δ9-THC, Δ8-THC, their hydroxymetabolites, cannabinol, cannabidiol, cannabichromene, cannabicyclol and olivetol which shares the C-ring conformation of these cannabinoids were all shown to impair thymidine, uridine and leucine incorporation into lymphocytic DNA, RNA and protein long ago [53]. Δ9-THC and olivetol were shown to increase lymphocytic chromosomal segregation errors and the number of hypodiploid cells [53].

When normal human lymphocytes from adult volunteers were incubated with micromolar concentrations of Δ9-THC a significant increase in chromosomal segregation errors was observed [53]. A higher number of chromosomal bridges, anaphase lags, micronuclei, unequal segregations in bipolar divisions and multipolar divisions was noted. The difference in anaphase lags and unequal divisions was significant [53]. The photomicrographs of many chromosomal bridges in telophase nuclei in [53] are very dramatic indeed. Unequal divisions presage the heightened incidence of chromosomal trisomies and monosomies noted epidemiologically in the Introduction. Anaphase lag is a precursor lesion to micronucleus formation which is the primary engine for chromothripsis and chromoanagensis and micronuclei have long been associated with cannabinoid exposure [65].

Cannabis has been known to be positive in the micronucleus assay which is one of the primary genotoxicity assays for over fifty years [65, 66]. Micronuclei have been shown to form when a chromosome becomes derailed and disconnected from the main mitotic spindle during the anaphase separation of the chromatids [67]. This is thought to be due to the impact of cannabis to interfere with the tubulin subunits of the microtubular arms of the mitotic spindle and with the actin subunits of the cellular cytoskeleton [67–69]. Lacking the normal complement of the many enzymes involved in gene maintenance and transcription, the genetic material becomes pulverized and then re-annealed in a haphazard manner as a result of the normal processes of gene transcription particularly on the lagging strand of DNA. This process thus gives rise to chromothripsis [67, 68].

Prenatal cannabis use was linked with Downs syndrome in offspring in an Hawaiian study published in 2007 [70], with Downs syndrome in Colorado, Canada and Australia [71–74] and more recently with Downs syndrome, Trisomies 13 and 18 and the monosomy Turners syndrome in the USA [75]. PCE has also been linked with Deletion 22q11.2 in the USA in spatiotemporal analyses and in odds ratio-based assessments [75].

Downs syndrome is known to greatly elevate the ALLR from around 3/100,000 to about 5 / 100 [76, 77]. Downs syndrome ALL is a B-Cell ALL RUNX1 positive disorder involving a translocation between chromosomes 12 and 21 [64].

Cannabis use is also linked with chromosome 12 pathophysiology. Testicular cancer invariably involves oncogenic licensing of chromosome 12 usually as an isochromosome 12p with reduplication of the short arms but alternately intrachromosomal gene amplification has also been described. All four studies examining the link between cannabis use and testicular cancer have been positive [78–81], and three have shown a dose-response relationship [78, 79, 81].

ALL has been described as usually resulting from protooncogene formation and re-arrangements due to translocations between various chromosome combinations including chromosomes 12 and 21 (ETV6-RUNX1), 4 and 11 (MLL-AF4), 1 and 19 (E2A-PBX1), 9 and 22 (BCR-ABL1), trisomy 4 and 10, ETV6-RUNX1-like, DUX4-rearranged, hyperploidy, hypoploidy, and intra-chromosomal rearrangements of chromosome 21 [7]. Interestingly MLL is also known as KMT2a (histone lysine methyltransferase 2a) [64] and both it and RUNX1 have major epigenomic activities.

#### Epigenomic pathways

It is established that ALL cells are mostly B-lymphocytes precursors which are arrested in their cellular differentiation and are therefore said to experience a “differentiation block”. The epigenetic machinery carried on or near DNA controls the expression of the genes. Hence the epigenetic state controls tissue specificity of cells and their differentiation stage by controlling factors such as DNA methylation, histone methylation and acetylation and post-translational modifications generally, micro-RNA expression, long non-coding RNA expression, the availability of enhancers to promoters, the activity of non-coding DNA and repeat segments, 3-D position of chromatin within the nucleus, proximity to topologically active domains or transcriptionally active gene “factories” and similar factors [82]. Moreover perturbations of epigenomic control can lead to genetic lesions and reciprocally genetic lesions can induce epigenomic changes [82].

It is important to observe that the genome of embryonic stem cells and precursor cells generally is largely demethylated and more open in its chromosomal conformation making it much more susceptible to genomic and epigenomic insults than the adult genome [83].

In relation to ALL both MLL / KDM2a and RUNX1 (also known as acute myeloid leukaemia protein 1) are key components of the epigenetic machinery. There are 28 million CpG islands in human DNA and their methylation state to a large extent controls the activation of the promoter regions of genes. Methylation of promoter DNA is a key step in leukaemogenesis [83] and several of the leukaemic fusion proteins are epigenomic effectors and change the DNA methylation state globally [63]. RUNX1 directly controls the state of differentiation of haemopoietic precursor cells.

Cannabinoids themselves carry a large epigenomic footprint. THC has been shown to reduce the level of synthesis of nuclear histones, sometimes by half [84, 85]. Marked epigenomically-mediated reduction of brain D2-dopamine receptors has been demonstrated in F1 rodent offspring following PCE [86]. Marked genome wide alteration in nucleus accumbens DNA methylation status has also been shown in another study of rodent F1 offspring after PCE [87–89]. This was replicated recently by a coordinated study of rodent F1 offspring and rat and human sperm [90]. And epigenetically mediated alteration in TH1 and TH2 lymphocyte proliferation in an F1 generation within lymph nodes has also been shown [91].

### Metabolism

Epigenomic modifications of both DNA and histones require small molecules produced from intermediate metabolism such as methyl, acetyl and sumoyl groups. Therefore any process which inhibits cellular metabolism can secondarily perturb the epigenomic state.

Importantly the mitochondria contain 16 KB of their own DNA which carries the code for some of their proteins. Therefore healthy cellular function requires that the genome of the mitochondria and that of the cell nucleus have coordinated expression of their genomic material. This is known as mitonuclear balance and is mediated both by small molecule metabolites and by malate-aspartate, glycerol-3-phosphate and nicotinamide mononucleotide shuttles and some extra-nuclear sirtuins including sirtuin 2 [92].

In this regard fumarate and succinate are known as oncometabolites and their corresponding disorders, fumarase deficiency and succinate dehydrogenase mutations are known to predispose to malignancy and cause germ line mutations as they slow the tricarboxylic acid cycle and interfere with the supply of metabolic substrates to the epigenetic machinery [82, 93].

### Cannabinoids and mitochondria

For these reasons it is highly pertinent that cannabinoids inhibit mitochondrial metabolism by many pathways. It is not widely known that the outer mitochondrial membrane of mitochondria carry all the signalling apparatus of the plasmalemma for the reception and transduction of cannabinoid signals [94–100]. The mitochondrial outer membrane carries cannabinoid type 1 receptors (CB1R’s) [95, 96]. This makes sense as cannabinoids are lipid soluble and are easily able to traverse the plasmalemma.

Cannabinoids directly reduce the synthesis of many of the components of the electron transport chain including the F1 ATPase itself [69, 101]. Cannabinoids reduce the transmembrane potential and lower the proton gradient in many cell types [94, 97–100, 102]. They directly stimulate uncoupling protein 2 [98]. They slow many of the reactions of the tricarboxylic cycle and pyruvate dehydrogenase.

### Other pathways

Apoptosis is a calcium-dependent feed-forward process whereby release of calcium from endoplasmic reticulum stores precipitates massive dumping of calcium from mitochondrial stores which activates the nuclear caspases and other effectors of catastrophic DNA cleavage and cell death pathways [103, 104]. For this reason processes which interfere with calcium channels and calcium signalling make cells more resistant to apoptosis. Many oncoproteins in the leukaemic disease-cluster act in this manner [103, 104]. The vanilloid calcium channels TRPV4 and TRPV6 are implicated in this way [62]. Cannabinoids are known to act at TRPV1 and other vanilloid channels [105–108].

Ceramide signalling is known to be involved in apoptotic pathways [62, 103] and is a known target of cannabinoid signalling [109, 110].

Cyclic-AMP and adenyl cyclase are known to be key effectors of leukaemic cell apoptosis [62] and are primary targets of cannabinoid and addictive drug signalling generally [111].

Leukotrienes have been shown to increase oxidative stress and induce DNA damage and be pro-oncogenic [7] and cannabinoid actions via CB1R are well described as often being pro-inflammatory [112–117] including in lymphocytes [118]. This is relevant as heavy cannabis use in young adults is associated in many case reports with aggressive cancers developing at younger age [119–122]. A proinflammatory milieu causes endogenous retrotransposons (“jumping genes”) to jump and precipitates genomic instability [123–129]. This process releases repeat sequences of DNA into the cytoplasm where it triggers innate immunity pathways by the cGAS-STING pathway via interferon gamma [123, 127–130]. Once this is stimulated a powerful positive feed-forward loop is established whereby cell-intrinsic inflammation triggers further genomic instability and heightened inflammation. Hence this process has been linked with tumour aggressiveness and metastasis [130].

Indeed it has been suggested that ALL may be a preventable disease based on the association of immune and inflammatory pathways with its pathogenesis [6]. It is interesting to observe that this may in fact be actionable by a bold public health approach to control cannabis for the reasons outlined above.

Reports also exist of cannabinoids being anti-apoptotic by several mechanisms [131–133].

Hence it can be seen that there are many interfaces between cannabinoid, proinflammatory metabolic, mitochondrial and epigenomic pathways which are cancer relevant and make the epidemiologically observed link eminently biologically plausible.

### Ethnogenomics

Many tumours demonstrate significant differential rates by ethnic background. The biological basis of one such interaction was elegantly elucidated by research which traced such differential to a paradoxical activation of a P53 response element at position rs4590952 in the kit P53-RE on chromosome 9 which occurred only in light skinned races [134]. Three loci near this site have previously been identified in prior GWAS’s as conferring increased cancer vulnerability [135–137]. P53 is generally known as the guardian of the genome and P53 is widely connected across the genomic and epigenomic machinery of the cell to pause and halt genomic replication in the presence of genotoxic stress. However at this locus genomic stress has the paradoxical effect of inducing activation of genomic replication, apparently to induce the tanning response in the light skinned races and result in melanocyte replication and increased skin protection from ultraviolet light-induced carcinogenesis [134]. Since cannabinoid exposed cells are obviously genotoxically stressed this implies that in fair-skinned races genomic stress can paradoxically stimulate cell replication as implied here.

Other loci have since been described including rs995030 and this is an area of active research enquiry at this time [138].

Since both genetic background and cannabinoid exposure are key factors in determining ALLRs this strongly implies a gene-environment interaction.

### Generalizability

Study results are likely to be generalizable for several reasons. First, we utilize a large database from a populous nation. It would appear that the drug use and cancer incidence data are quite reliable, as are the population-based census data. It is also likely the most accessible dataset in the world relating drug use to ALL incidence. Secondly, we found similar results when data was interrogated by a variety of regression techniques. Thirdly, the results of the causal inference analysis are strongly positive with both inverse probability weighting and e-Value analyses being strongly confirmatory. Finally, findings satisfy eight of nine of Hill’s criteria of causality including strength of association, consistency amongst studies, specificity, temporality, coherence with other known data, biological plausibility, dose-response relationships and experimental confirmation [139]. Notwithstanding, as this relationship has not previously been reported elsewhere we feel that further replication in other contexts is important.

It is also noteworthy that study findings apply more broadly across the spectrum of cannabinoids than just implicating THC alone. Regression findings clearly implicated cannabigerol often more powerfully than THC. Positive and significant trends were observed for the bivariate relationship between ALLR and THC, cannabinol, cannabigerol, cannabichromene and cannabidiol. The action of cannabidiol and cannabidivarin to cause double stranded DNA breaks, micronucleus formation and directly oxidize all the bases of DNA, and slow protein DNA and RNA synthesis was noted earlier [19, 54–57, 59, 60].

### Strengths and limitations

This study has a number of strengths and limitations. Its strengths include the use of: a large population dataset and registry controlled data; a variety of advanced statistical methods including space-time regression, instrumental panel regression, and a number of robust and other regression models; spatially and temporally lagged models with robust results throughout; causal inference techniques including inverse probability weighting in multiple models and e-Value calculation; inclusion of a range of relevant potential covariates including other substance exposure, ethnicity, income and the intensity of use of cannabinoids by various ethnicities; and use of well-validated multiple imputation techniques to examine the effects of ethnic differentials in ALLR. The principal limitation of this study relates to the non-availability of individual patient level substance use data, a limitation which is common to most epidemiological studies of this kind. Indeed because of recall bias, and because individual participants may be confused about whether their pregnancy was cannabinoid affected after cessation of cannabis exposure early in the pregnancy, we advocate the development of a robust biomarker, possibly derived from epigenomic or glycomic analyses as has previously been advanced [140].

## Conclusion

Study data show for the first time that pediatric ALLRs are robustly related to state-level cannabis exposure and to ethnic THC exposure. Prominent dose-response and quintile effects are demonstrated with marked effects of cannabis legalization. Results are confirmed at space-time regression and shown to be causal by techniques of causal inference particularly inverse probability weighting and e-Values, which are all strongly confirmatory. Cannabis legalization was associated with significantly higher ALLRs both when legal status was considered and when dichotomized legal status was reviewed. In so doing we greatly extend prior work, show that the cannabis-ALL link is salient at the population health level, is likely a primary driver of the 93.5% monotonic rise in ALLRs since 1975, and is a primary contributor to the well described ethnic differentials in ALL incidence, likely related to differential intensity of cannabis exposure and strongly suggesting a gene-environment interaction. Such results are therefore pivotal in re-focussing the pediatric cancer discussion on substance use and cannabinoid exposure in particular. In that ALL is the commonest malignant disorder of the pediatric age group, the present results leave open the possibility that increasing cannabis exposure is a key driver of the marked increases in total pediatric cancers since 1975. Findings implicate all cannabinoids examined including THC, cannabigerol, cannabichromene, cannabinol and cannabidiol. In that ALL is well described as being due to formation of several protooncogenes and oncoproteins by a series of chromosomal translocations the present clear results add an important mechanistic dimension to the trisomy / monosomy series of defects previously described in association with prenatal cannabis use in addition to anaphase chromosomal mis-segregation [68, 75]. Since pediatric cancer is known to be related to gestational genetic and epigenetic defects these transgenerational impacts add a further major dimension to the cannabis legalization debate which has not been widely considered [141, 142]. Future research directions could include study of this relationship at higher geotemporal resolution and in other contexts and with sensitive objective biomarkers of cannabinoid exposure [140].

## Supplementary Information

**Additional file 1.**

## Data Availability

No permissions are required to access the data which was used and collated in this study, e.g. NSDUH study. Data including shapefiles and R programming script is made publicly available on the Mendeley Data Archive at this URL: 10.17632/cf8c43yv62.1 .

## Abbreviations

ABL1: Tyrosine protein kinase ABL protooncogene-1
ACOG: American College of Obstetricians and Gynaecologists
AF4: AF4 lymphoid nuclear protein
AIAN: American Indian Alaska Native
ALL: Acute Lymphoid Leukaemia
ALLR: Acute Lymphoid Leukaemia Rate
AUD: Alcohol Use Disorder
BCR: B-Cell Receptor
cAMP: Cyclic Adenosine Monophosphate
CB1R: Cannabinoid Type I Receptor
CD19: B-lymphocyte antigen
CD3: Cluster of differentiation 3
CDC: Centres for Disease Control, Atlanta, Georgia
cGAS: Cytoplasmic Guanosine-Adenosine Synthase
DNA: Deoxyribonucleic acid
DUX4: Double Homeobox 4
E2A: Basic helix-loop-helix binding factors E2A
ETV6: Translocation-Ets-leukaemia virus 6
e-Values: Expected values
F1ATPase: F1- ATP synthase (inner mitochondrial membrane)
KMT2a: Histone Lysine N-Methyltransferase 2A
lambda: Spatial autocorrelation coefficient
MICE: Multiple Imputation by Chained Equations
MLL: Mixed Lineage Leukaemia
NHPI: Native Hawaiian Pacific Islanders
NSDUH: National Survey of Drug Use and Health
P53: Tumour protein P53
P53-RE: P53-Response Element
PBX1: Pre-B-cell leukaemia transcription factor 1
PCE: Pre-natal cannabis exposure
PCED: PC-esterase domain containing
phi: Idiosyncratic component of the spatial error term
psi: Individual time-invariant component of the spatial error term
RDAS: Restricted Data Archive System
rho: Spatial autoregressive parameter
RNA: Ribonucleic Acid
RUNX1: Runt-related transcription factor 1
SAMHDA: Substance Abuse Mental Health Data Archive
SAMHSA: Substance Abuse Mental Health Service Administration
SEER: Surveillance Epidemiology and End Results, from CDC
sf: Simple Features
STING: Stimulator of Interferon Genes
THC: Δ9-Tetrahydrocannabinol
