# Supplementary material for: Cannabinoid exposure as a major driver of pediatric acute lymphoid Leukaemia rates across the USA: combined geospatial, multiple imputation and causal inference study
Source: BMC Cancer. 2021 Sep 3;21:984. doi: 10.1186/s12885-021-08598-7 (PMC8414697; doi:10.1186/s12885-021-08598-7)
Supplement: Supplementary file 1 — Additional file 1. [file 12885_2021_8598_MOESM1_ESM.pdf]

ST1 - Kriged ALL Data

State	Year																
	2001	2002	2003	2004	2005	2006	2007	2008	2009	2010	2011	2012	2013	2014	2015	2016	2017	Total
Alabama	1.6	1.7	1.3	2	1.2	1.5	1.6	2	1.6	1.6	1.7	2.2	1.7	2	2	2.5	1.9	1.8
Arkansas	2.2	1.6	2.3	1.4	1.5	1.3	1.7	1.8	2.3	1.7	1.8	1.9	2.1	1.9	2.2	2.5	1.8	1.9
Arizona	3	2.8	2.3	2.2	2.4	2.5	2.3	2.5	3	2.5	2.4	2.3	2	2.3	3.4	2.6	2	2.5
California	2.5	2.5	2.4	2.7	2.5	2.8	2.8	2.9	2.9	2.9	2.7	2.7	2.7	3	3	3	2.7	2.8
Colorado	2.7	1.9	2.5	2	1.9	2.2	2.6	2.5	2.9	1.7	2.8	2	2.2	1.9	2.1	2.4	2	2.3
Connecticut	1.8	2.4	2.1	2.7	2.9	2.2	2.6	2.4	2.8	2.4	2	2	1.6	1.7	1.5	1.5	2.6	2.2
Florida	2.5	2.2	2.2	2	2.1	2.1	2.6	2.8	2.1	2.5	2.2	2.5	2.4	2.3	2.6	2.4	2.5	2.4
Georgia	2.1	1.9	1.9	1.6	1.8	2	2	1.5	2.2	1.9	2.2	1.7	1.5	1.7	1.7	2	2.1	1.9
Idaho	2.2	2.2	2.2	2.2	2.2	2.8	2.5	2.2	2.2	2.6	2.9	2.2	2.4	2.1	3	2.3	1.9	2.2
Illinois	2.1	1.9	2.2	2.4	2.2	2.5	2.1	2.4	2.1	2.2	2.2	2.2	2.3	2	2.2	2.4	2.3	2.2
Indiana	2.1	1.5	1.7	2.1	2.3	1.9	2.2	2.5	1.9	2.4	2.2	2.6	1.8	1.6	2.5	2.3	1.4	2.1
Iowa	1.9	2.4	1.7	2	1.9	1.6	2.2	1.6	2.7	2	2.3	1.9	2.3	1.9	2.6	3.2	2.2	2.1
Louisiana	1.8	1.7	1.4	1.6	1.7	1.4	2.4	1.7	1.6	1.6	2.2	1.5	1.7	1.6	2.3	1.4	1.6	1.7
Maryland	1.3	1.6	1.5	1.5	1.3	1.2	1.6	1.9	1.9	1.6	1.7	1.6	1.7	1.5	1.7	2.1	1.8	1.6
Michigan	2.1	2.3	1.8	1.8	2.1	2.1	1.7	2.1	2.1	2.3	2.4	2	2.1	2.1	1.8	1.9	1.7	2
Minnesota	2.5	1.6	2.2	2.7	2.1	2	2	2.3	2	2.2	2.4	2.5	2.5	1.6	2.4	1.7	2.5	2.2
Missouri	2.1	1.4	2.4	1.6	2.2	1.8	1.8	1.7	2.2	2	1.9	1.8	1.7	1.9	1.9	2.2	1.4	1.9
Mississippi	1.8	1.8	1.7	1.1	2	1.7	2.1	2.1	1.4	1.3	1.6	1.9	1.9	1.9	2.1	1.8	1.7	1.8
North Carolina	2.1	1.3	1.8	1.7	2	1.9	1.9	2	2.3	2.4	2	2.2	1.9	2.1	2.2	2	1.7	2
Nebraska	1.9	2.6	3.3	2.1	2.05	2	1.93	1.87	1.8	2.6	2.7	2.5	2.5	2.5	2	2.3	2.7	2.1
New Jersey	2.2	2.1	2.2	2.4	2.5	2.5	2.7	1.8	2.5	2.4	2.2	2.2	2	2.6	2.2	2.8	2.5	2.3
New Mexico	3.7	3.5	2.3	3.6	2.8	2.9	2.1	2.2	2.2	2.2	2.7	2.1	3	2.5	3	3	2.3	2.7
Nevada	2.8	1.5	1.6	2.8	2.1	2.3	2	2	2.7	3.1	3.1	1.2	2.3	2.4	2.3	3.3	2.2	2.3
New York	1.9	2.2	2	2.3	2.1	2.1	2.2	2.4	2.3	2.4	2.4	2.4	2.1	2.4	2.3	1.8	2	2.2
Ohio	1.6	1.9	2	2.3	1.8	2	1.8	1.9	1.9	2.1	2.3	1.7	2	2	2.1	2.3	1.7	2
Oklahoma	2.3	1.9	2.2	1.8	2	2.5	2.1	2.1	2	1.9	2.1	2.3	2	1.2	2.4	2.2	1.6	2
Oregon	2.8	2.4	1.7	2.1	2.1	2.8	3.2	2.6	2.3	1.9	2	2.9	2	2.4	3.3	2	2.6	2.4
South Carolina	1.5	2.4	1	2.4	1	1.5	2.3	1.6	1.7	2.1	2.3	1.9	1.7	1.6	1.9	1.8	1.9	1.8
Tennessee	1.8	1.5	1.7	2.7	2.1	2.1	2.1	2.1	1.9	1.9	2.4	2.3	2	2	2.2	1.7	2.4	2
Texas	2.5	2.3	2.2	2.4	2.3	2.5	2.5	2.6	2.7	2.5	2.5	2.5	2.5	2.4	2.6	2.6	2.6	2.5
Utah	1.2	2.2	2.1	2.1	2.7	1.7	2	2.6	1.4	1.8	2.7	2.4	2.7	1.9	2.1	2.3	2.7	2.2
Virginia	1.3	1.8	1.8	1.9	1.9	2.1	2	2.4	1.6	2	2.1	1.6	1.3	1.8	2.1	1.8	1.6	1.8
Washington	2	2.1	1.8	2.5	2.1	2.4	2.6	2	2.3	1.9	2.1	2.4	2.6	2.7	1.8	2.2	2.1	2.2
Wisconsin	1.7	2.3	2.2	1.8	2.3	2.6	2.1	2.6	2.4	2.5	2	2.4	2.1	1.9	1.8	1.9	1.1	2.1
